# PDZD8 interacts with Protrudin and Rab7 at ER-late endosome membrane contact sites associated with mitochondria

**DOI:** 10.1038/s41467-020-17451-7

**Published:** 2020-07-20

**Authors:** Yael Elbaz-Alon, Yuting Guo, Nadav Segev, Michal Harel, Daniel E. Quinnell, Tamar Geiger, Ori Avinoam, Dong Li, Jodi Nunnari

**Affiliations:** 1 Department of Molecular and Cellular Biology, University of California Davis, Davis, CA USA; 2 National Laboratory of Biomacromolecules, CAS Center for Excellence in Biomacromolecules, Institute of Biophysics, Chinese Academy of Sciences, Beijing, China; 3 College of Life Sciences, University of Chinese Academy of Sciences, Beijing, China; 4 Department of Biomolecular Sciences, Weizmann Institute of Science, Rehovot, Israel; 5 Department of Human Molecular Genetics and Biochemistry, Tel-Aviv University, Tel Aviv-Yafo, Israel; 6 Present Address: Department of Molecular Genetics, Weizmann Institute of Science, Rehovot, Israel

**Keywords:** Endosomes, Endoplasmic reticulum, Mitochondria

## Abstract

Endosomes are compositionally dynamic organelles that regulate signaling, nutrient status and organelle quality by specifying whether material entering the cells will be shuttled back to the cell surface or degraded by the lysosome. Recently, membrane contact sites (MCSs) between the endoplasmic reticulum (ER) and endosomes have emerged as important players in endosomal protein sorting, dynamics and motility. Here, we show that PDZD8, a Synaptotagmin-like Mitochondrial lipid-binding Proteins (SMP) domain-containing ER transmembrane protein, utilizes distinct domains to interact with Rab7-GTP and the ER transmembrane protein Protrudin and together these components localize to an ER-late endosome MCS. At these ER-late endosome MCSs, mitochondria are also recruited to form a three-way contact. Thus, our data indicate that PDZD8 is a shared component of two distinct MCSs and suggest a role for SMP-mediated lipid transport in the regulation of endosome function.

## Abstract (web summary)

Membrane contact sites between organelles have been shown to play important biological roles. Here, the authors show that at the ER, PDZD8 associates with Protrudin and also with Rab7 endosomes and recruits mitochondria to form three-way contacts.

## Introduction

The endoplasmic reticulum (ER) is a vast organelle that extends throughout the cell and has a central role in protein biogenesis, and lipid and ion homeostasis. To conduct these functions, the ER forms physical contacts with essentially all other membranous organelles through membrane contact sites (MCSs). MCSs are regions where the membranes of two organelles are brought into close apposition (10–30 nm) by a proteinaceous tether to form subdomains that specialize in non-vesicular lipid transfer and ion transfer, and function as signaling hubs and in the control of organelle dynamics^1–4^.

Several types of ER MCSs between the ER and membranes of the endocytic pathway have been identified^5–7^. The endocytic pathway functions to control the fate of material that enters cells via endocytosis and thus governs many cellular and physiological processes, including immunity, signaling, nutrient availability, and organelle quality control^8^. The membrane compartments that arise from the fusion of endocytic vesicles are collectively known as endosomes, which undergo a process of maturation and specialization from early to late endosomes and to lysosomes to promote protein sorting and degradation^8^. Endosomal maturation requires the sequential recruitment and activation of Rab GTPases, which function as molecular switches that cycle between the GDP and GTP bound state in a spatially and temporally regulated manner via GTPase-activating proteins and guanine nucleotide exchange factors. The Rab switch involves the coordinated dissociation of Rab5 on early endosomes and recruitment and activation of Rab7, which drives the biogenesis of late endosomes via interactions with its large number of interactors/effectors. The Rab switch is coupled to endosomal acidification and lipid composition remodeling, including PIP lipids, which transition from an early endosome enrichment of PI(3)P to PI(3,5)P2 and recruit specific subsets of proteins to drive endosomal maturation^8,9^. PI(3)P synthesis is mediated by the Beclin1-Vps15-Vps34 complex (PI3K complex), which is recruited by Rab5^10,11^. As endosomes mature, PI(3)P is converted into PI(3,5)P2 by the PIKFYVE kinase that is recruited through its PI(3)P-binding FYVE-domain, and phosphatases of the myotubularin (MTM) family are also recruited via a FYVE-domain to deplete PI(3)P^12,13^. In addition, PIP conversion is regulated by the Rab7 effector, WDR91, which is recruited to late endosomes, where it inhibits PI3K activity, preventing the generation of PI(3)P, to promote early to late endosomal maturation^14^.

ER-endosome MCSs have emerged as regulators of endosomal function and behavior, including endosomal dynamics, positioning, and motility^5,7,15,16^ and the propensity to form ER-endosome MCS increases as endosomes mature^17^. Several components of ER-endosome MCSs have been described. These include members of the family of ER integral VAMP-associated proteins (VAPs), which participate in diverse ER-MCSs via their ability to interact with proteins containing a short FFAT motif^5,6,15^. VAPs interact with multiple proteins at the ER-endosomal membrane contact site, including the cholesterol transfer proteins STARD3^18^ and ORP1L^19,20^, which sense and regulate the flow of cholesterol to and from the ER via non-vesicular transport activity. ER-late endosome MCSs also serve to integrate the motility of late endosomes on microtubules with cellular status, such as cholesterol levels, by regulating their association with the plus and minus end motors, kinesin-1 and dynein, respectively^15,19–22^. Rab7 controls this bidirectional endosome movement through the recruitment of adapters for dynein and kinesin-1, RILP and FYCO1, respectively^19,23,24^. At ER-late endosome MCSs, under conditions of low cholesterol, cytosolic ORP1L engages with GTP-Rab7 on late endosomes and VAPs at the ER and negatively regulates the association of late endosomes with the minus end Dynein microtubule motor complex^20,21^. ER-endosomal MCSs also positively regulate plus-ended microtubule endosomal transport via the ER protein Protrudin. Protrudin localizes to endosomal membranes via interactions with both endosomal PIP lipids and Rab7, where it also interacts with VAPs via an FFAT motif^22,25^. At ER-late endosomes MCSs, Protrudin is thought to promote the association of kinesin-1 with FYCO1 to mediate transport of late endosomes to plus end of microtubules^22^. Thus, a picture is emerging in which a diverse set of ER-endosome MCSs create microdomains at the endosomal surface that integrate cellular status with the spatiotemporal regulation of endosomal dynamics, maturation, and motility^3,15,26^.

Here we report an ER-late endosomal contact site comprised of the multi-domain integral ER proteins, PDZD8, which is a Synaptotagmin-like Mitochondrial lipid-binding Proteins (SMP) domain-containing protein, and Protrudin, and the late endosomal Rab GTPase, Rab7. Structure-function analysis indicates that distinct domains of PDZD8 mediate interactions with Protrudin and Rab7 and that PDZD8 and Protrudin are independently recruited to ER-late endosome MCSs. PDZD8 was previously identified as a component of an ER-mitochondria MCS, although the identity of its mitochondrial partner is not known^27^. Consistent with this, we also observe that mitochondria can be recruited to PDZD8-enriched ER microdomains at ER-late endosome MCSs to form a three-way contact. Our observations are consistent with the idea that ER-late endosomal contact sites are dynamic plastic structures that regulate endosomal behavior.

## Results

### PDZD8 binds Protrudin and is recruited to late endosomes

**Fig. 1:**
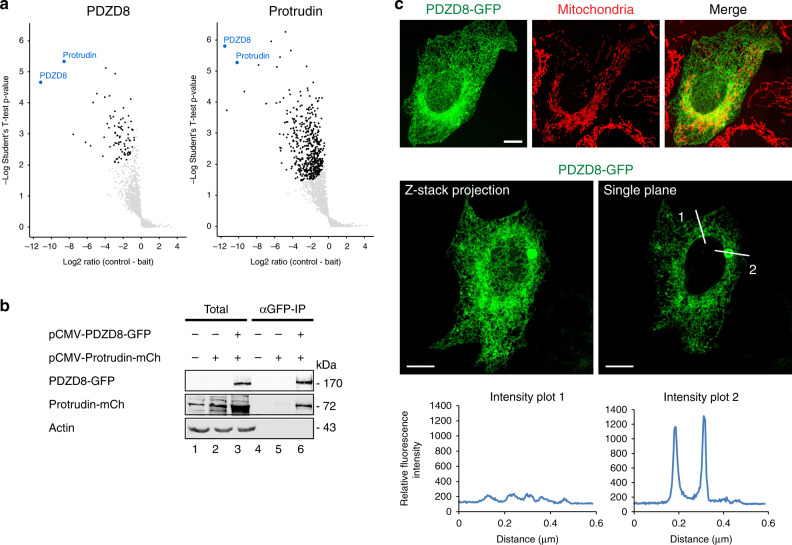
PDZD8 interacts with Protrudin and is enriched in ER subdomains. **a** Endogenous PDZD8 and Protrudin interact. Volcano plots of mass-spectrometry based results from an analysis of immunoprecipitates using anti-PDZD8 (left panel) and anti-Protrudin (right panel) antibodies from crosslinked human HCT116 cell lysates showing significant potential interactors (in black), identified using one-sided Student’s *t* test (FDR 0.05, S0 = 0.1). PDZD8 and Protrudin are labeled in blue. Volcano plots represent experiments performed in three biological replicates for each condition. Source data are provided as a Source Data file. **b** Analysis of the PDZD8-GFP and Protrudin-mCherry interaction by immunoprecipitation from cell extracts. Extracts from HEK293T cells expressing Protrudin-mCherry, both Protrudin-mCherry and PDZD8-GFP or with no vector were immunoprecipitated with anti-GFP-Trap beads and western blot analysis was performed using anti-GFP, anti-Actin, and anti-Protrudin antibodies for detection of PDZD8-GFP, Actin, and Protrudin-mCherry (designated Protrudin-mCh), respectively. Source data are provided as a Source Data file. **c** PDZD8-GFP localizes to ER and to ER subdomains in cells. Top panel: representative images of U2OS cells expressing PDZD8-GFP (z-stack projection). Pdzd8-GFP (in green) and mitochondria (in red) labeled with MitoTracker DeepRed. Scale bar: 10 μm. Middle panel: U2OS cell expressing PDZD8-GFP (in green) shown full Z-stack projection (left) and a single plane (right). Lower panel: plots of pixel intensity versus distance using ImageJ software corresponding to the lines marked 1 and 2 on the single plane image in the middle panel.

The ER protein, PDZD8, was recently identified as an ER- mitochondria membrane contact site protein, however, the molecular nature of this MCS is not known^27^. To address this and further investigate the mechanism of action of PDZD8, we identified interacting proteins by mass spectrometry based analysis of endogenous PDZD8, isolated from crosslinked human HCT116 cell extracts using validated anti-PDZD8 antibodies^28^. Mass spectrometry analysis of crosslinked endogenous PDZD8 immunopurifications revealed that the predominant interactor was Protrudin, an integral ER protein previously shown to reside at ER-late endosome MCSs^22^ (Fig. 1a, left panel; list provided in Supplementary Dataset 1). Consistently, mass spectrometry analysis of reciprocal immunopurifications of endogenous Protrudin from crosslinked cell extracts using a validated anti-Protrudin antibody revealed that its predominant interactor was PDZD8 (Fig. 1a, right panel; list provided in Supplementary Dataset 2). Immunoprecipitation and western analysis of extracts from HEK293 cells co-overexpressing PDZD8-GFP and Protrudin-mCherry also indicated that these components interact, which further validated the PDZD8-Protrudin interaction detected using antibodies directed against the endogenous tag-less versions of these proteins (Fig. 1b). Thus, our data indicate that PDZD8 and Protrudin interact in cells.

**Fig. 2:**
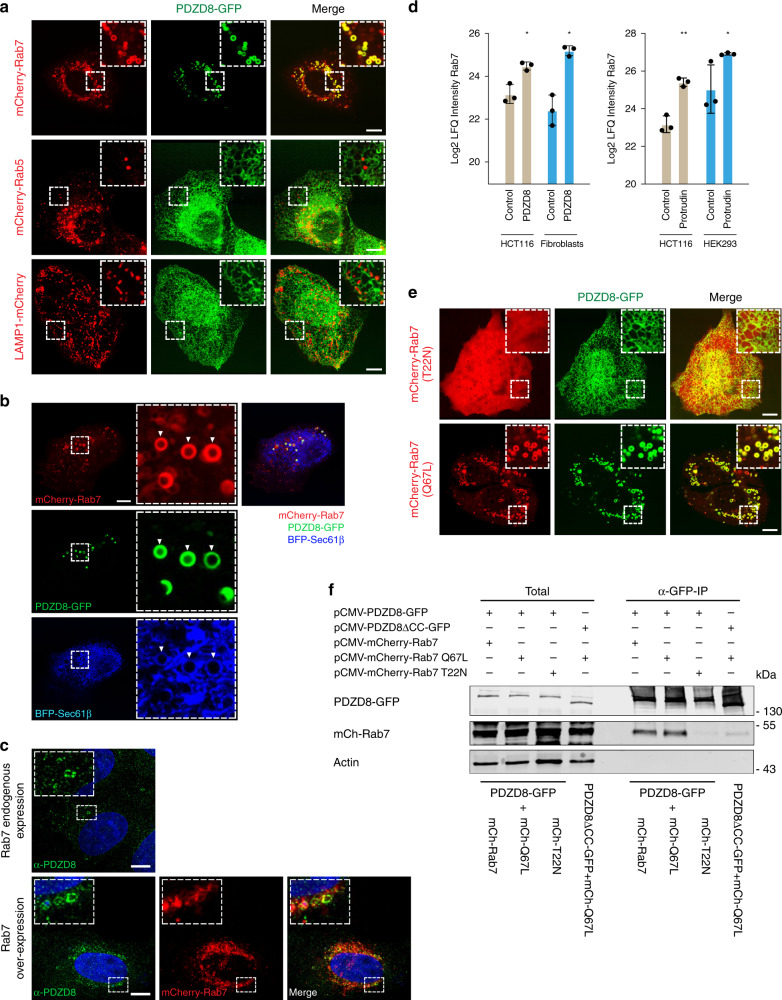
PDZD8-GFP interacts with GTP-Rab7. **a** PDZD8 co-localizes with late endosomes. Representative images of U2OS cells expressing PDZD8-GFP and mCherry-Rab7 (late endosomes), mCherry-Rab5 (early endosomes), or LAMP1-mCherry (lysosomes). Scale bar: 10 μm. **b** PDZD8-GFP is enriched in ER subdomains associated with late endosomes. U2OS cells expressing PDZD8-GFP (green), mCherry-Rab7 (red), and BFP-Sec61β (blue). Arrowheads indicate areas where the BFP-Sec61β-labeled ER co-localized with PDZD8-GFP associated with mCherry-Rab7 endosomes. Scale bar: 10 μm. **c** Endogenously expressed PDZD8 is recruited to Rab7-late endosomes. Immunofluorescence analysis of U2OS cells (upper panel) versus U2OS cells expressing mCherry-Rab7 (red). Cells were fixed, incubated with an anti-PDZD8 (green) and secondary anti-rabbit AlexaFluor488 antibodies and stained with Hoechst stain (blue, nucleus). Scale bar: 10 μm. **d** Rab7 is enriched in native PDZD8 and Protrudin immunoprecipitates. Mass-spectrometry analysis of Rab7 in immunopurifications of PDZD8 from crosslinked HCT116 cell extracts and human fibroblasts (left), and of Protrudin from crosslinked HCT116 cells and HEK293 cells (right). Rab7 intensity values are log2 label-free quantification (LFQ). One sided Student *t* test was performed between the control (no antibody) and indicated antibody with permutation-based FDR *q* value < 0.05 and S0 = 0.1. * and ** refer to FDR *q* value <0.05 and 0.01, respectively. Data presented as mean values ± S.D. Three biological replicates were performed. *P*-values: 0.0045 (FDR *q*-value 0.044; test statistic −3.477) and 0.0015 (FDR *q*-value 0.0497; test statistic −1.139) for PDZD8 vs control; HCT116 and fibroblasts, respectively. *P*-values: 0.0007 (FDR *q*-value 0.006; test statistic −5.797) and 0.0326 (FDR *q*-value 0.0486; test statistic −2.223) for Protrudin vs control; HCT116 and HEK293, respectively. Source data are provided as a Source Data file. **e** Rab7-GTP is required for recruitment of PDZD8-GFP to late endosomes. U2OS cells co-expressing PDZD8-GFP and mCherry-tagged Rab7 T22N (top) and Q67L (bottom) mutants. Scale bar: 10 μm. **f** Analysis of PDZD8-GFP and Rab7 interaction by immunoprecipitation. Extracts from HEK293T cells expressing PDZD8-GFP or PDZD8ΔCC-GFP, and wildtype mCherry-Rab7 or mCherry-Rab7 T22N or Q67L mutants were immunoprecipitated using anti-GFP-Trap beads and western analysis was performed using anti-GFP, anti-Actin and anti-Rab7 antibodies for detection of PDZD8-GFP, Actin, and mCherry-Rab7 respectively.

Published work indicates that Protrudin localizes to ER- late endosome MCSs primarily via its PIP lipid-binding FYVE domain, where it functions to facilitate the regulation of endosomal motility^22,29^. Given the stable interaction we observed between Protrudin and PDZD8, we considered whether PDZD8 was also recruited to endosomal MCSs. To address this question, we transiently transfected GFP-tagged PDZD8 and performed live-imaging of GFP-tagged PDZD8 (PDZD8-GFP) in human U2OS cells. For all live-cell imaging of transiently expressed tagged constructs, we transfected cells with the minimal amount of plasmid sufficient for microscopy detection to avoid protein overexpression artifacts. Under these conditions, we estimate that PDZD8-GFP was ~10-fold overexpressed compared to endogenous protein (Supplementary Fig. 1). PDZD8-GFP primarily localized diffusely in the ER (Fig. 1c; whole-cell projection), however, at a lower frequency, we also observed PDZD8-GFP localized at a significantly higher intensity to spherical structures, suggesting an enrichment of PDZD8 at specific ER subdomains (Fig. 1c; single plane image). We co-overexpressed PDZD8-GFP with mCherry tagged markers of early endosomes (Rab5), late endosomes (Rab7), and lysosomes (LAMP1) to test whether these PDZD8-enriched subdomains were associated with endosomes. PDZD8-GFP did not co-localize with either Rab5 or LAMP1-labeled endosomal structures (Fig. 2a). In contrast, in cells co-expressing PDZD8 and Rab7, we observed that PDZD8-GFP spheres co-localized with mCherry-Rab7-labeled endosomes and that there was a significant increase in the number of PDZD8-GFP labeled spheres per cell, as compared to the diffusely ER-localized and relatively rare PDZD8-GFP spheres observed in cells overexpressing PDZD8 alone (compare Fig. 1c with Fig. 2a, Supplementary Fig. 2, 2.5 ± 1.5 per cell compared to 65.7 ± 27.4 per cell, *n* = 20 cells). To test whether Rab7-colocalized PDZD8 spheres reflected the recruitment of PDZD8-GFP enriched ER co-localized with late endosomes, we imaged cells co-expressing PDZD8-GFP and mCherry-Rab7 and the general ER marker, BFP-Sec61β. Consistently, at regions of co-localization of PDZD8-GFP with mCherry-Rab7-labeled endosomes, we also observed BFP-Sec61β-labeled ER spheres tightly associated with late endosomes (Fig. 2b). Although the increased number of PDZD8-enriched ER subdomains associated with late endosomes was dependent on overexpressing both PDZD8 and Rab7 (Figs. 1c and 2a, and Supplementary Fig. 2), endogenous PDZD8 in U2OS cells, as detected by indirect immunofluorescence of PDZD8, using a validated anti-PDZD8 antibody^28^, was also observed in spherical structures, and in cells overexpressing Rab7-mCherry PDZD8- structures colocalized with Rab7-labeled endosomes (Fig. 2c and Supplementary Fig. 3). In addition, we observed that Rab7 peptides were significantly enriched in immunopurifications from crosslinked cellular fractions of endogenous PDZD8 and Protrudin, (Fig. 2d). These data suggest that ER-associated PDZD8 is recruited to late endosomes in a Rab7-dependent manner.

Rab proteins serve as molecular switches that are active while bound to GTP and are inactivated upon GTP hydrolysis to GDP. Activated GTP-bound Rab7 is recruited to the late endosome membrane and recruits additional proteins to the membrane that act as effectors and control endosomal maturation and motility^9^. We further tested the role of Rab7 in recruiting PDZD8-enriched ER domains to endosomes by assessing the guanine nucleotide specificity of Rab7 in PDZD8 endosome recruitment. ER-localized PDZD8 was recruited to endosomes in cells expressing the constitutively GTP-bound Rab7Q67L, but not the GDP-bound Rab7T22N (Fig. 2e). We also assessed the Rab7 guanine nucleotide specificity by examining the interaction between PDZD8 and Rab7 by immunoprecipitation in cells overexpressing PDZD8-GFP and different versions of Rab7: only Rab7 and the GTP-bound Rab7Q67L, and not the GDP-bound Rab7T22N, were detected by western analysis of anti-GFP immunoprecipitates from cell extracts, consistent with our cytological data (Fig. 2f). Together these data indicate that ER-localized PDZD8 is recruited to late endosomes in a manner dependent on GTP-activated Rab7. These observations are consistent with a recent study published while this work was in review reporting that PDZD8 and Rab7-GTP are enriched at ER-late endosome contact sites^30^.

### Distinct PDZD8 domains bind Protrudin and late endosomes

**Fig. 3:**
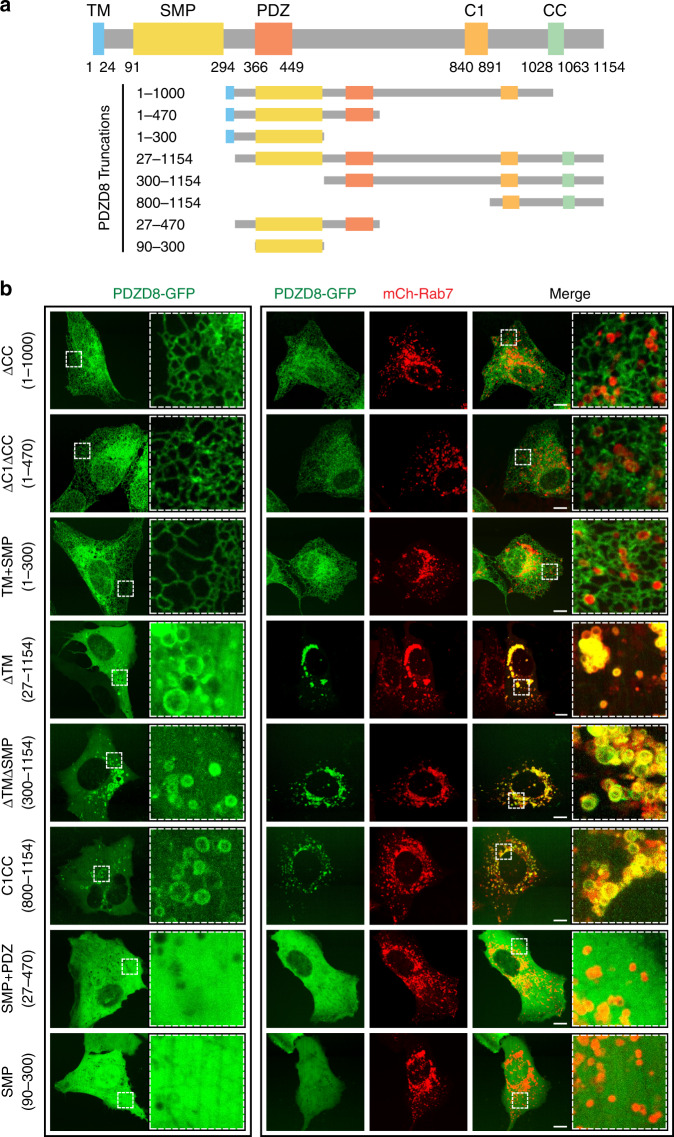
The PDZD8 C1 and CC domains are sufficient for recruitment to Rab7-endosomes. Domain analysis of the interaction of PDZD8 with Rab7-endosomes. **a** Schematic diagram depicting PDZD8 domain structure. TM transmembrane, SMP synaptotagmin-like mitochondrial lipid-binding proteins, PDZ postsynaptic density protein (PSD95), Drosophila disc large tumor suppressor (DlgA) and Zonula occludens-1 protein (ZO-1), C1 protein kinase C conserved region 1, CC coiled-coil. **b** GFP-tagged truncated versions of PDZD8 that were analyzed. Representative images of U2OS cells expressing GFP-tagged truncated versions of PDZD8 alone (left panel) or with mCherry-Rab7 (right panel). Scale bar: 10 μm.

We exploited the increased number of PDZD8-enriched ER subdomains associated with late endosomes under conditions of co-overexpression of PDZD8 and Rab7 to robustly determine the PDZD8 domains important for ER recruitment to endosomes. Given that PDZD8 is a multidomain containing protein, we generated and analyzed truncated versions of PDZD8-GFP with different domain compositions (Fig. 3a, schematic). As expected, expression of truncated constructs containing the single N-terminal transmembrane (TM) domain were localized to the ER in cells whereas constructs lacking the TM were localized to the cytosol (Fig. 3b, ΔCC:1-1000, ΔC1ΔCC:1-470 and TM + SMP:1-300 versus ΔTM:27-1154, ΔTMΔSMP:300-1154, C1CC:800-1154, SMP + PDZ:27-470, and SMP:90-300, respectively). However, in the absence of the TM, constructs containing the C1 and coiled-coil domains were also localized to spherical structures in cells, similar to those observed at low frequency in cells overexpressing full length PDZD8-GFP (Figs. 1c and 3b, ΔTM:27-1154, ΔTMΔSMP:300-1154, C1CC:800-1154). In contrast, constructs lacking the TM and the C1 and coiled-coil domains, were strictly localized in a diffuse manner in the cytosol (Fig. 3b, SMP + PDZ:27-470, SMP:90-300). Expression of either the C1:800-1000 or CC:1000-1154 regions was not detected in cells. Our observations suggest that the C1CC:800-1154 portion of PDZD8 is sufficient for PDZD8 recruitment to late endosomes. Consistent with this, we observed that PDZD8 C1CC:800-1154 co-localized with mCherry-Rab7-labeled endosomes in cells (Fig. 3b). In addition, the CC domain of PDZD8 was also necessary for the recruitment of PDZD8 to Rab7-labeled late endosomes, as a construct lacking the CC domain localized diffusely to the ER membranes in cells under conditions of Rab7 overexpression (Fig. 3b, ΔCC:1-1000). Consistent with this, we observed a decrease in the efficiency of co-immunoprecipitation of PDZD8ΔCC with Rab7Q67L by western blot analysis (Fig. 2f). Thus, our data suggest that recruitment of an ER PDZD8 subdomain to late endosomes is mediated via the PDZD8 C terminal C1/CC regions in a GTP-Rab7-dependent manner.

**Fig. 4:**
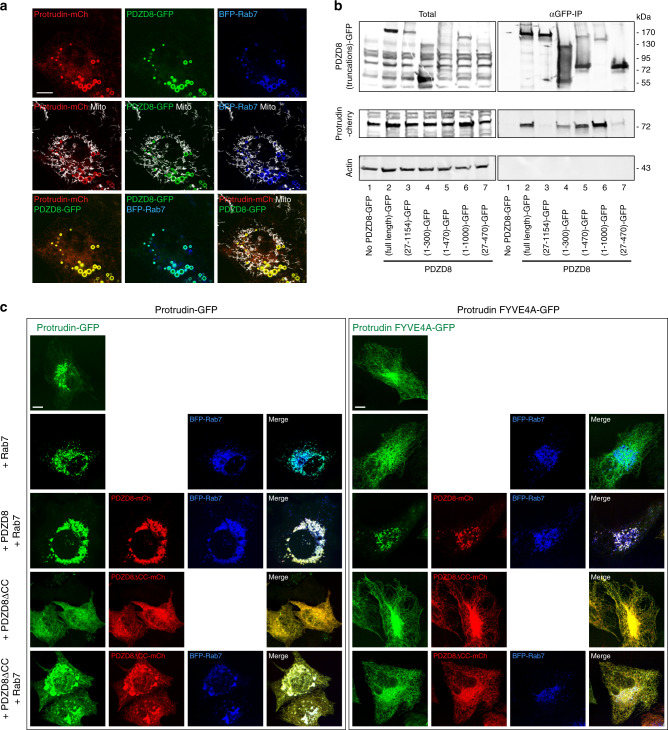
PDZD8 and Protrudin associate independently with late endosomes. **a** PDZD8-GFP co-localizes with Protrudin-mCherry on BFP-Rab7-labeled late endosomes. Representative image of a U2OS cell co-expressing PDZD8-GFP, BFP-Rab7, and Protrudin-mCherry. Mitochondria were labeled with MitoTracker DeepRed. Scale bar: 10 μm. **b** The PDZD8 TM domain is required for its interaction with Protrudin. Extracts from HEK293T cells expressing various GFP-tagged truncated versions of PDZD8 (as illustrated in Fig. 3a) and Protrudin-mCherry were immunoprecipitated with GFP-Trap beads and western blot analysis was performed using anti-GFP, anti-Actin anti-Protrudin antibodies for detection of PDZD8-GFP truncated versions, actin and Protrudin, respectively. Source data are provided as a Source Data file. **c** PDZD8-GFP and Protrudin-mCherry are independently recruited to BFP-Rab7-labeled endosomes. Representative images of PDZD8 KO HeLa cells expressing either wildtype Protrudin-GFP or Protrudin FYVE4A-GFP and co-expressing BFP-Rab7 with either PDZD8-mCherry or PDZD8ΔCC-mCherry. Scale bar: 10 μm.

Our results indicate that endogenous PDZD8 and Protrudin interact (Fig. 1a). Protrudin has been previously shown to reside at ER-late endosome MCSs^22^. To test whether ER-localized PDZD8 and Protrudin are recruited together to late endosomes in Rab7-dependent manner, we co-expressed BFP-Rab7, PDZD8-GFP, and Protrudin-mCherry in cells. Consistently, both PDZD8-GFP and Protrudin-mCherry were co-localized with Rab7-labeled late endosomes (Fig. 4a). Thus, our results suggest that PDZD8, Protrudin, and Rab7 interact together at ER subdomains associated with late endosomes in cells. We tested which domains of PDZD8 are required for its interaction with Protrudin using anti-GFP immunoprecipitations from HEK293T cells co-expressing PDZD8-GFP constructs (Fig. 3a, schematic) and Protrudin-mCherry (Fig. 4b). Consistent with the observed reciprocal interactions of endogenous PDZD8 and Protrudin in immunoprecipitates (Fig. 1), full-length overexpressed PDZD8-GFP efficiently co-immunoprecipitated with Protrudin-Cherry (Fig. 4b, compare no PDZD8-GFP, lane 1 with (full length)-GFP, lane 2) from cell extracts. In contrast, PDZD8 constructs lacking the TM domain were not efficiently co-immunoprecipitated with Protrudin from cell extracts (Fig. 4b, compare (full length)-GFP, lane 2 with (27-1154)-GFP and (27-470-GFP), lanes 3 and 7, respectively). The PDZD8 construct containing only the N-terminal TM and SMP domains was also efficiently co-immunoprecipitated with Protrudin (Fig. 4b compare (full length)-GFP, lane 2, with (1-300)-GFP, lane 4). Given that this construct lacks the C1 and CC regions that were sufficient for the recruitment of PDZD8 to endosomes (Fig. 3b), this observation suggests that PDZD8 utilizes distinct domains for interacting with Protrudin and GTP-Rab7-labeled late endosomes. These data also suggest that the PDZD8-Rab7-endosome interaction occurs in a Protrudin-independent manner: the PDZD8 construct lacking the TM domain failed to co-immunoprecipitate with Protrudin, but retained its ability to be recruited in a Rab7-dependent manner to the late endosomal membrane (Figs. 3b and 4b, compare (full length)-GFP, lane 2, with (27-1154)-GFP, lane 3) and the PDZD8 1-1000 lacking the C-terminal CC domain efficiently co-immunoprecipitated with Protrudin, but failed to be recruited to endosomes in a Rab7-dependent manner (Figs. 3b and 4b, compare (full length)-GFP, lane 2, with (1-1000)-GFP, lane 6).

To more directly test whether PDZD8 and Protrudin are independently recruited to late endosomes, we examined the localization of Protrudin-GFP in a previously characterized HeLa PDZD8 knock-out (KO) cell line generated using CRISPR-CAS9^28^. Consistent with independent targeting pathways, Protrudin-GFP was efficiently recruited to Rab7-labeled endosomes in PDZD8 KO cells (Fig. 4c, left top two panels). Consistent with published data^22^, in the absence of PDZD8, the recruitment of Protrudin to Rab7-late endosomes was primarily dependent on its PIP-binding FYVE domain, as Protrudin-FYVE4A-GFP, in which alanine replaced each of the four basic residues within the FYVE domain, localized diffusely to the ER in PDZD8 KO cells (Fig. 4c, right top two panels). This observation indicates that in PDZD8 KO cells, overexpression of Rab7 alone is not sufficient to recruit Protrudin-FYVE4A-GFP to late endosomes. However, in PDZD8 KO cells expressing BFP-Rab7 and PDZD8-mCherry, Protrudin-FYVE4A-GFP co-localized to Rab7-endosomes (Fig. 4c, compare right top two panels with right (+PDZD8 + Rab7) panel). This PDZD8-dependent mode of Protrudin-FYVE4A-GFP recruitment to BFP-Rab7-labeled endosomes required the PDZD8 CC region, consistent with our data indicating a role for CC domain in the recruitment of PDZD8 to late endosomes (Figs. 3b and 4c, compare right top two panels with right (+PDZD8ΔCC + Rab7) panel). We also observed that Protrudin-GFP was not as efficiently recruited to BFP-Rab7-labeled endosomes in PDZD8 KO cells expressing PDZD8 lacking the CC region, as Protrudin-GFP was localized diffusely to the ER (Fig. 4c, compare left top three panels with left (+PDZD8ΔCC) and (+PDZD8ΔCC + Rab7) panels). This observation is consistent with our immunoprecipitation data indicating that PDZD8 lacking the CC region retained its ability to interact with Protrudin at the ER membrane (Fig. 4b, lane 6). Collectively, these results support a model where, at the ER membrane, PDZD8 and Protrudin interact and influence each others recruitment to late endosomes, but each can also be independently targeted to ER-late endosomes subdomains via Rab7-GTP and PIP lipids, respectively.

### Mitochondria are recruited to PDZD8-Rab7 contact sites

**Fig. 5:**
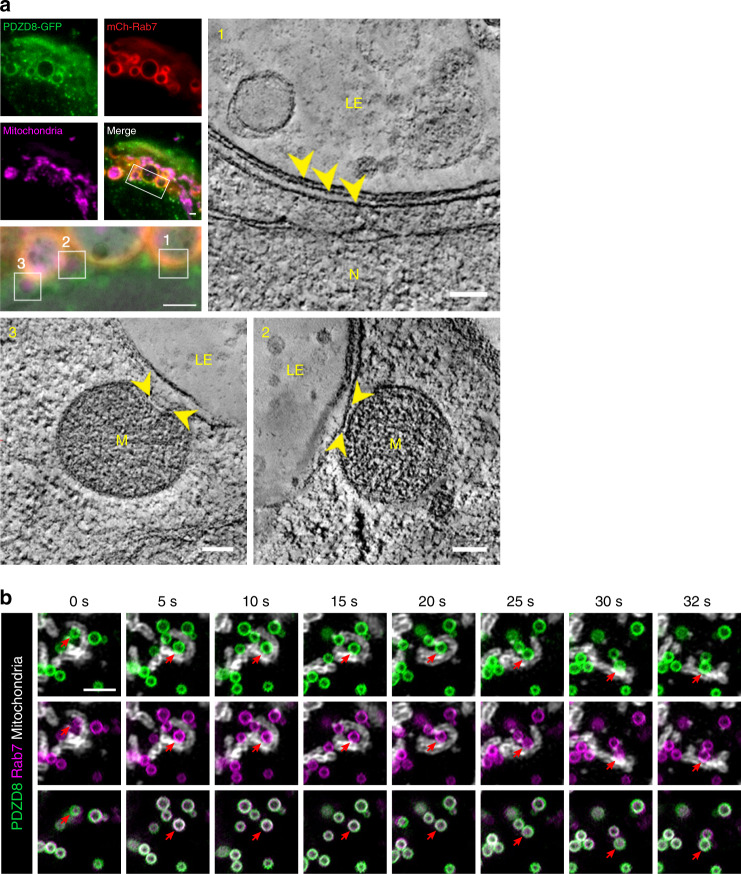
Mitochondria associate with PDZD8- Rab7 labeled ER-endosome contacts. **a** Correlative light and electron microscopy (CLEM) analysis indicates regions co-labeled with Rab7 and PDZD8 in cells are contacts between ER, late endosomes and mitochondria. Upper left panel: In-resin fluorescence images of U2OS cells expressing PDZD8-GFP (green), mCherry-Rab7 (red, mCh-Rab7) and MitoTracker DeepRed (magenta). Rectangle area on merged image corresponds to the magnified image under the panel (left middle), which is an overlay of the transmission electron micrograph and in-resin fluorescence images. Scale bars: 1 μm. Boxed regions, labeled 1–3 correspond to slices from 3D electron tomograms (upper and lower right and left images). Arrowheads in (1) indicate three membrane bilayers corresponding the late endosome membrane and two membranes of an ER tubule; Scale bar: 100 nm. Measured distance between the membranes of ER and LE is ~15 nm. Arrowheads in (2–3) mark the proximity of mitochondria to the ER-late endosome contact site. The CLEM experiment was repeated twice from independent samples of transiently transfected cells. TEM tilt-series for tomographic reconstruction were acquired from three individual cells that exhibited fluorescence of all three markers (PDZD8-GFP, mCherry-Rab7 and MitoTracker DeepRed). Scale bar: 100 nm. Late endosome (LE), mitochondria (M), and nucleus (N). **b** Mitochondria are persistently localized with late endosomes in contact with PDZD8 ER subregions. Time lapse GI-SIM imaging of BFP-Rab7 labeled endosomes in cells expressing PDZD8-GFP. Red arrow indicates a PDZD8-Rab7 mediated ER-LE-mitochondria contact site. Representative images from eight independent GI-SIM imaging sessions. Scale bar: 2 μm.

Membrane contact sites are unique cellular structures mediated by proteins in adjacent organelle membranes that tether organelles in close proximity (estimated 10–30 nm apart) to enable non-vesicular transfer of lipids and solutes. To test whether the PDZD8-GFP ER subdomains colocalized with Rab7-labeled endosomes are bona-fide membrane contact sites, we analyzed regions of co-localization at high resolution using correlated light and electron microscopy (CLEM). In areas where PDZD8-GFP and mCherry-Rab7 were co-localized, as inferred from the fluorescence signals, we observed regions of ER in contact with late endosomes, as evidenced by the presence of multivesicular bodies (Fig. 5a inset 1). In addition, the distance between the ER and late endosome membrane measured ~15 nm, characteristic of a membrane contact site (Fig. 5a, inset 1, arrow heads). Thus, our data indicate that the PDZD8-GFP ER subdomains associated with late Rab7-labeled endosomes represent ER-endosome membrane contact sites.

In many regions of PDZD8-GFP and mCherry-Rab7 co-localization, where we observed an ER-late endosome contact site, we also observed a mitochondrion (Fig.5a, inset 2), whose shape was co-altered with associated ER adjacent to an endosome (Fig. 5a, inset 3). This observation suggests that PDZD8-late endosomal MCSs may recruit mitochondria in cells. To further test this possibility, we assessed the proximity of mitochondria to Rab7-labeled endosomes in U2OS cells overexpressing PDZD8-GFP or PDZD8ΔCC-GFP, which either contain or lack PDZD8 ER subdomains localized to Rab7-labeled endosomes, respectively (Fig. 3b). Mitochondrial proximity was quantified by the number of overlaps observed between thresholded images of mCherry-Rab7 labeled late endosomes and mitochondria, labeled using Mitotracker Deep Red FM (Thermo Fisher Scientific; Supplementary Fig. 4, left and right panels, respectively, representative thresholded images, *n* = 8 cells for each condition). Based on this analysis, cells expressing PDZD8-GFP, had a higher fraction of the Rab7-labeled endosomes in proximity to mitochondria as compared to cells expressing PDZD8ΔCC-GFP (Supplementary Fig. 4, bottom panel).

We also performed Grazing Incidence Structured Illumination Microscopy (GI-SIM) super resolution live-cell imaging and examined the relative movement of PDZD8-labeled ER and Rab7-labeled endosomes. Analysis of movement of Rab7-labeled endosomes and the ER using either the general ER marker mEmerald-Sec61β, PDZD8-GFP or PDZD8ΔCC-GFP revealed that PDZD8-GFP, but not PDZD8ΔCC-GFP overexpression, significantly decreased endosome motility relative to endosomes in cells expressing mEmerald-Sec61β, consistent with our observation that co-overexpression of PDZD8 and Rab7 results in the formation of multiple highly associated ER-endosomal regions in cells (Supplementary Fig. 5, temporally color-coded map over 50 s, upper panel: mEmerald-Sec61β; middle panel: PDZD8-GFP; lower panel: PDZD8ΔCC-GFP, and corresponding Supplementary Movies M1–M3). In addition, GI-SIM imaging also indicated that PDZD8-labeled ER subdomains associated with Rab7-labeled endosome were stably localized adjacent to mitochondria (Fig. 5b, arrows, and Supplementary Movie M2), consistent with CLEM analysis (Fig. 5a) and proximity analysis (Supplementary Fig. 4). In total, our data suggest that overexpression of PDZD8 and Rab7 facilitates the formation of contacts between both ER-endosomes and ER-mitochondria in the context of a three way MCS, consistent with previous work indicating that PDZD8 is a component of an ER-mitochondria tether^27^.

### The Rab7 effector, WDR91, marks PDZD8-associated endosomes

**Fig. 6:**
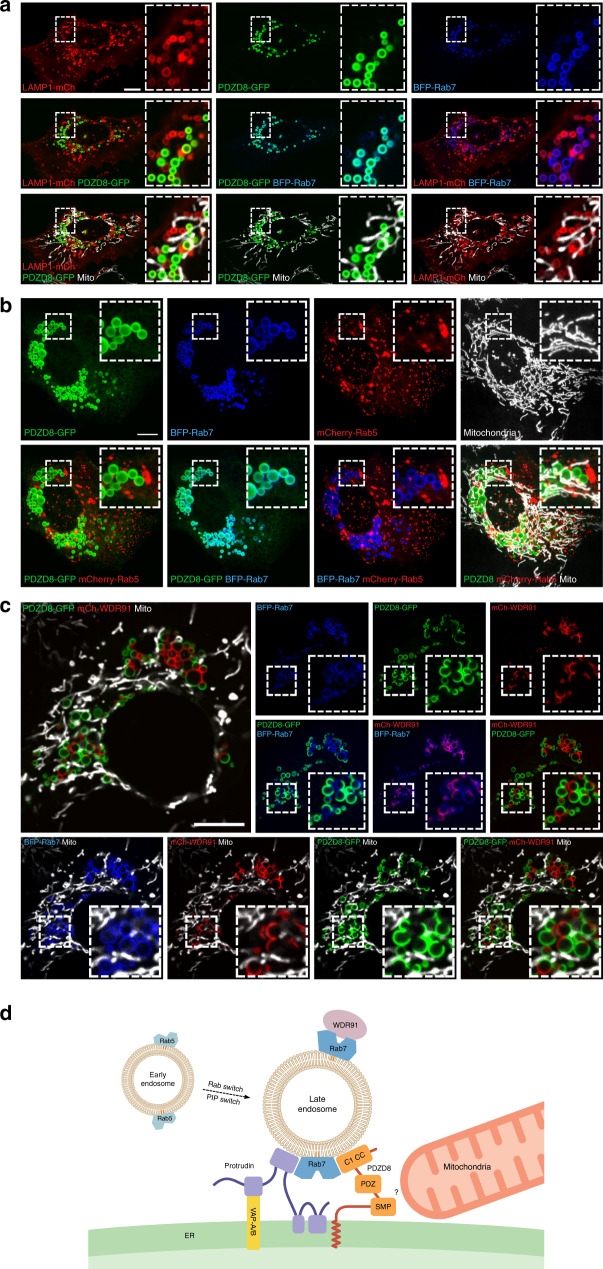
PDZD8-Rab7 mediated ER-LE contact site can form during endosomal maturation. **a** The lysosomal marker LAMP1 is not detected in PDZD8-associated Rab7-lableled endosomes in cells Live imaging of U2OS cells co-expressing PDZD8-GFP, BFP-Rab7, and LAMP1-mCherry. Mitochondria are labeled with MitoTracker DeepRed. Inset corresponds to magnified area. Scale bar: 10 μm. **b** The early endosomal marker, Rab5, is not detected in PDZD8-associated Rab7-labeled endosomes in cells. U2OS cells expressing PDZD8-GFP (in green), BFP-Rab7 (in blue), and mCherry-Rab5 (in red). Mitochondria are labeled with MitoTracker DeepRed (in white). Scale bar: 10 μm. **c** The Rab7 effector, WDR91 localizes to PDZD8-labeled late endosomes. U2OS cells co-expressing PDZD8-GFP (in green), BFP-Rab7 (in blue), and mCherry-WDR91 (in red). Mitochondria are labeled with MitoTracker DeepRed (in white). Inset corresponds to magnified area. Scale bar: 10 μm. **d** Schematic representation of the PDZD8/Protrudin-Rab7 ER-endosome membrane contact site.

The early to late endosome maturation process consists of an intertwined complex series of steps, which include endosomal acidification, a Rab5 to Rab7 switch, an interdependent conversion of membrane PIP from PI(3)P to PI(3,5)P2 and an alteration of the lipid composition as late endosomes become depleted in phosphatidylserine (PS) and rich in the unique phospholipid lysobisphosphatidic acid (LBPA)^31–33^. To gain insight into what endosomal maturation stage the PDZD8-mediated ER-late endosome MCS forms, we co-expressed in U2OS cells PDZD8-GFP and BFP-Rab7 with either mCherry-Rab5 or LAMP1-mCherry. LAMP1 is a membrane protein that is targeted from the Golgi to lysosomes through a lysosomal targeting motif^34^. Rab7 and LAMP1 have been shown to co-localize on late endosomes and lysosomes^9^. In cells overexpressing both PDZD8 and Rab7, however, Rab7-labeled endosomes that possessed regions of associated PDZD8-labeled ER were not labeled by LAMP1 (Fig. 6a). When PDZD8 and Rab7 were co-expressed with the early endosomal marker Rab5, the endosomal population that was labeled by PDZD8 and Rab7 also showed no overlap with Rab5 labeled early endosomes (Fig. 6b). We next co-expressed PDZD8-GFP and BFP-Rab7 with mCherry-WDR91, a Rab7 effector that controls the phosphoinositide conversion step in endosomal maturation and serves as a marker for the PIP conversion step^35^. When co-expressed in cells, WDR91 and PDZD8 were localized together on late endosomes uniformly labeled with Rab7 in a mutually exclusive pattern (Fig. 6c), which has not been previously observed for WDR91^35^. The localization of WDR91 and PDZD8 to the same population of late endosomes suggests that the PDZD8 ER-endosome MCS may form at an endosomal maturation stage during the PIP conversion step in which the Rab5 to Rab7 switch is complete.

Although we do not know the mechanism underlying the mutually exclusive localization pattern of PDZD8-GFP and mCherry-WDR91 associated with Rab7-lableled endosomes, we exploited it to resolve the localization of mitochondria relative to the PDZD8 ER subdomains associated with Rab7-labeled endosomes. In cells co-expressing BFP-Rab7, mCherry-WDR91, and PDZD8-GFP, mitochondria were observed more frequently in close proximity to the PDZD8-associated portion of Rab7-endosomes (Fig. 6c, bottom panel). We analyzed the fraction of total BFP-Rab7 and Mitotracker pixels that overlapped, and then calculated how many of these pixels also overlapped with either PDZD8-GFP or mCherry-WDR91 pixels (Fig. S6, magenta colored pixels in left and right panels, respectively). This analysis revealed that a majority of the contact area between Rab7-labeled-endosomes and mitochondria occurs adjacent to PDZD8-GFP co-localized with Rab7-labeled endosomes (Supplementary Fig. 6, bar graph), consistent with our previous observations (Fig. 5). GI-SIM live imaging of cells expressing mCherry-WDR91, BFP-Rab7, and PDZD8-GFP supported this conclusion as mitochondria are persistently localized over time to portions of Rab7-endosomes that are associated with PDZD8-ER subdomains (Supplementary Movie M4). Thus, these data support the conclusion that mitochondria localize with PDZD8 ER subdomains associated with Rab7-labeled endosomes in cells.

## Discussion

The ER is the most extensive eukaryotic endomembrane system, spreading throughout the cell and connecting with virtually all other organelles via MSCs, which function as hubs to control and coordinate organellar functions with cell status^4^. Our results identify the ER membrane proteins, PDZD8 and Protrudin, together with the late endosomal protein Rab7, as components of an ER-late endosome MCS in mammalian cells. In cells, overexpression of PDZD8 and Rab7 is sufficient to increase the number contacts between the ER and late endosomes (Fig. 2 and Supplementary Fig. 2), suggesting that PDZD8 and Rab7 are parts of an active MCS tether, as defined by the community of scientists working on membrane contact sites^36^. Our data suggest a model where this ER-late endosome contact site is created via an interaction between the C1 and CC regions of the ER-anchored PDZD8 and GTP-Rab7 (Figs. 3 and 6d). The interaction we observe between ER membrane-associated PDZD8 and Protrudin is independent of the PDZD8 C1 and CC regions. Thus, our data support a model in which PDZD8 utilizes its distinct domains to separately interact with Rab7-endosomes and with Protrudin. Although PDZD8 can recruit Protrudin to Rab7-endosomes, Protrudin is also independently recruited to endosomes via its FYVE region, presumably via an interaction with PI(3)P^22^. We speculate that this redundant, but interdependent PDZD8-Protrudin-Rab7-late endosome interaction network serves to form a highly specific and regulatable ER-late endosome MCS. Several additional ER-late endosome contact sites proteins have been described. Both cytosolic ORP1L that is recruited to late endosomes through its interaction with GTP-Rab7^20,21^ and the endosomal membrane proteins STARD3/STARD3NL^18^ contain FFAT motifs that enable their binding to the ER tail-anchored proteins, VAP-A/B. Another proposed ER-late endosome tether is composed of the ER protein ORP5 and the endosomal membrane protein NPC1^37^. The relative roles each of these presumably distinct tethers play at the ER-late endosome remains an outstanding question.

Future work is required to understand the functional role of the ER PDZD8-Protrudin/Rab7-endosome MCS. One possibility is that this MCS functions to promote endosomal maturation. This is potentially supported by the co-localization of the Rab7 effector WDR91, which facilitates the conversion of PI(3)P to PI(3,5)P during maturation, on the population of PDZD8/Protrudin/Rab7-labeled endosomes, which also lack LAMP1, a marker that localizes to Rab7 endosomes at a later maturation stage. The PDZD8-mediated ER-late endosome MCS forms selectively with activated GTP-Rab7, which suggests that it is dynamic, and temporally regulated during maturation. Maturing endosomes undergo dramatic changes in membrane composition such as the depletion of phosphatidylserine from late endosomes and the enrichment of LBPA^32,33^. PDZD8 is one of only six proteins in the human proteome that harbors a Synaptotagmin-like Mitochondrial lipid-binding Proteins (SMP) domain. SMP domain proteins characterized to date have been shown to localize exclusively to membrane contact sites in human cells, as well as in yeast^38,39^ and structural analyses indicate a general role of SMP domain proteins in interorganellar phospholipid transfer^39^. Thus, it is possible that PDZD8 functions in phospholipid transfer, similar to all characterized SMP domain proteins, to promote endosomal maturation. Protrudin is thought to control late endosome motility by facilitating the transfer of kinesin-1 from the ER to late endosomes^22^. Our mass spectrometry analysis of immunopurified endogenous Protrudin supports previous reports of its interactions with kinesin-1 and the VAP family proteins (Fig. 1a and Supplementary Dataset 2). Thus, it is also possible that ER PDZD8-Protrudin/Rab7-endosome MCS functions to regulate late endosomal motility and distribution, which is intimately linked to endosomal maturation.

PDZD8 has been suggested recently to mediate the formation of ER-mitochondria contact sites^27^, although the mitochondrial partner is yet unknown. Our data are consistent with PDZD8 as an ER-mitochondrial tether: high resolution CLEM, and fluorescence microscopy (FM), including super-resolution GI-SIM analyses suggest that mitochondria are recruited to the PDZD8 ER subdomain associated with Rab7-endosomes. The significance of this observation at present is not clear. It is possible that the relatively high concentration of PDZD8 on the ER subdomain associated with Rab7 endosomes observed under our over-expression conditions drives via mass action the interaction with mitochondria. Alternatively, but not exclusively, the endosome may somehow be required for the formation of a PDZD8-dependent ER-mitochondria contact, which would suggest a role for mitochondria in endosomal function/maturation. It is also possible that PDZD8’s lipid transfer domain may function as a conduit for phospholipid transfer between the ER, endosomes, and mitochondria to drive endosomal maturation. Our observation of two contact sites serving to link three organelles suggests a mechanism for interorganellar communication.

## Methods

### Mammalian cell growth and transfection

U20S and HEK293T cells (ATCC) were grown in high-glucose Dulbecco’s Modified Eagle’s Medium (DMEM) supplemented with 10% fetal bovine serum (FBS) and 1% penicillin/streptomycin. Human fibroblasts were cultured in RPMI medium supplemented with 20% fetal bovine serum (FBS) and 1% penicillin/streptomycin. HCT116 cells (ATCC) were grown in McCoy’s 5A medium supplemented with 10% fetal bovine serum (FBS) and 1% penicillin/streptomycin. For imaging, U2OS cells were seeded at ~0.5 × 10^5^ cells per ml 24 h prior transient transfection in 35-mm glass-bottom imaging dishes (MatTek). Plasmid transfections were performed for 5 h in reduced serum medium (Opti-MEM^TM^, ThermoFisher) with Lipofectamine 2000 transfection reagent (ThermoFisher). Cells were imaged 18 h post transfection in DMEM supplemented with 10% FBS. All DNA plasmids used in this work are listed in Supplementary Table 1. DNA primers used for generating constructs reported in this work are listed in Supplementary Table 2.

### Antibodies

Primary antibodies: anti- beta Actin (Abcam Ab8224; 1:2000), anti-PDZD8 [Zhang 2015, 1:2500], anti-Protrudin (ProteinTech 12680-1-AP; 1:2000), anti-GFP (Abcam Ab290; 1:2000), anti-Rab7 (Cell Signaling Technology D95F2; 1:2000). Secondary antibodies: Goat anti-Rabbit lgG H&L 680 (Abcam Ab216777; 1:10000), Goat anti-Mouse lgG H&L 680 (Abcam Ab216776; 1:10000), IRDye 800CW Goat anti-Rabbit lgG (LI-COR Biosciences 926-3211; 1:10000), IRDye 680RD Goat anti-mouse lgG (LI-COR Biosciences 926-68070; 1:10000), Alexa Fluor 488 goat anti-Rabbit (ThermoFisher Scientific A-11034; 1:1000), and Alexa Fluor 568 goat anti-Mouse (ThermoFisher Scientific A-11031; 1:1000).

### Immunofluorescence

Cells were seeded as described above; 24 h later, cells were stained with 100 nM MitoTracker™ Deep Red FM (ThermoFisher) for 15 min at 37 °C. Cells were washed once in complete medium and fixed in 4% paraformaldehyde in Phosphate-buffered saline (PBS) pH 7.4 for 20 min at room temperature. Cells were then washed twice in PBS and permeabilized in PBS supplemented with 0.1% TritonX-100 for 20 min. Cells were incubated in 3% bovine serum albumin (BSA) PBS solution for 1 h at room temperature prior to the addition of primary antibodies at 1:1000 dilution in PBS supplemented with 1% BSA and 0.1% Tween-20 overnight at 4 °C, rinsed twice in PBS, and incubated with Alexa-Fluor–conjugated secondary antibodies at 1:2000 dilution in same solution for 1 h. Antibodies used: goat anti-mouse AlexaFluor 568 conjugate and goat anti-rabbit AlexaFluor 488 conjugate (ThermoFisher).

### Spinning-disc confocal microscopy

Live-cell imaging was performed using VisiScope Confocal Cell Explorer system, composed of a Zeiss Yokogawa spinning disk scanning unit (CSU-W1) coupled with an inverted Olympus IX83 microscope. Cells were imaged at 37 °C, under 5% CO_2_. Images were acquired using a ×100 oil lens and captured by a connected PCO.Edge sCMOS camera, controlled by VisView software version 3.2.0.0 (Visitron Systems GmbH). If necessary, slight linear adjustments to contrast and brightness were made using ImageJ 1.52 V (NIH). MitoTracker™ Deep Red FM (ThermoFisher M22426) was used to stain mitochondria.

Cells expressing either PDZD8-GFP or PDZD8-mCherry were imaged in >70 independent live-cell imaging experiments, cells expressing either Protrudin-GFP or Protrudin-mCherry were imaged in 55 different experiments and cells expressing PDZD8-GFP truncated constructs were each imaged in between 4 and 25 different experiments. The number of cells analyzed for each imaging condition was *n* > 100 and the images presented in the figures are representative.

### Immunopurifications and mass spectrometry analysis

Immunoprecipitation/mass spectrometry experiments were performed in three different human cell lines: HCT116, HEK293, and human fibroblasts, each with three biological replicates. Cells were grown in 15 cm dishes, harvested and washed in phosphate-buffered saline (PBS). Cells were re-suspended to 2 × 10^6^ cells/ml in PBS and the crosslinker dithiobis-succinimidyl-propionate (DSP) was added to a final concentration of 500 μM. Following 30 min of incubation at room temperature, Tris-Cl pH7.5 was added to a final concentration of 100 mM to quench the crosslinking. Cell pellets were re-suspended in lysis buffer (150 mM NaCl, 50 mM Tris-Cl pH7.5, 1% sodium-deoxycholate, 0.1% SDS, 1% NP-40, and 1 mM EDTA supplemented with protease inhibitors) and incubated on ice for 30 min. Lysates were centrifuged for 10 min in 8000×*g* at 4 °C and supernatant was collected and its protein concentration determined using Pierce™ BCA protein assay kit. In all, 10 mg of total protein per sample were diluted in lysis buffer to a 1-ml volume and used as starting material for each pull-down condition. In total, 2 μg of appropriate antibody were added to the lysate and incubated in constant rotation for 1 h at 4 °C, 100 μl mMACS™ protein G magnetic microbeads (Miltenyi Biotec) were added and incubated for 4 h at 4 °C. Columns were placed in the Multi™MACS M96 separator, equilibrated in lysis buffer, then pull-down samples were loaded onto columns and let flow. Columns were washed five times in 500 μl wash buffer (150 mM NaCl, 50 mM Tris-Cl pH7.5). Elution of samples from column was achieved through on-column trypsinization by 30 min incubation in 25 μl Elution buffer I (2 M Urea, 50 mM Tris-Cl pH7.5, 1 mM DTT, 5 mg/ml Trypsin), followed by the addition of 100 μl Elution buffer II (2 M Urea, 50 mM Tris-Cl pH7.5, 5 mM Chloroacetamide). Eluate was collected in a new tube and allowed continued trypsin digestion overnight. Peptide were acidified with TFA followed by purification on C18 stageTips^40^. Peptides were then loaded onto 50-cm-long EASY-spray PepMap columns (Thermo Scientific) and separated using a 140-min gradient of buffer A (0.1% formic acid) and 7–28% buffer B (80% acetonitrile, 0.1% formic acid) on the EASY-nLC1000 UHPLC system (Thermo Scientific) that was coupled to the Q-Exactive Plus or HF mass spectrometers (Thermo Scientific)^41^ via the EASY-Spray ionization source. All MS measurements were done in a data-dependent mode using a top-10 method. Data were acquired using Xcalibur software (Thermo Scientific). Raw MS files were analyzed with MaxQuant version 1.5.6.9 and the Andromeda search engine. MS/MS spectra were searched against the UniprotKB (version 2016_04) database.

All statistical tests and calculations were done using the Perseus software^42^ on the label free quantification (LFQ) intensity values after log2 transformation^43^. Data analysis was performed for each pull down separately after filtering for valid values in at least two samples of the pull down group. Data were imputed based on the assumption that missing values indicate low abundance; missing values were replaced with random values that create a Gaussian distribution with a downshift of 1.6 or 1.7 standard deviations and a width of 0.4 of the original distribution. To identify potential interactors one-sided Student’s *t* test was performed between the control and each pull down with permutation-based FDR *q* value <0.05 and S0 = 0.1^44^. The proteomic datasets that support these analyses are available in the PRoteomics IDEntifications (PRIDE) database via ProteomeXchange with identifier PXD015523.

### Sample preparation for correlative light and electron microscopy

Samples were prepared for electron microscopy as previously described in^45^ with minor modifications. In brief, U2OS cells (ATCC) were seeded on 3-mm carbon-coated sapphire disks (Wohlwend GmbH, Switzerland) and allowed to settle for 12–18 h. The cells were transfected as described above and fixed by high pressure freezing (HPF) using the Leica EM ICE (Leica Microsystems GmbH, Germany). For HPF, the cells were washed in FluoroBrite-DMEM media (Thermo Fisher Scientific) supplemented, with 20% FBS (Thermo Fisher Scientific), as cryo-protectant and placed between two aluminum planchettes (Wohlwend GmbH, Switzerland; Part: #242 and #389). Freeze-substitution and resin embedding were performed using a temperature-controlled device, AFS2 (Leica Microsystems GmbH, Germany) as previously described in McDonald et al.^46^. In brief, the HPF-fixed samples were placed in 0.05% (w/v) uranyl acetate in dry acetone at −90 °C for 10 h. The temperature was then raised to −45 °C (5 °C/h), and held for 5 h followed by three acetone washes. Infiltration with Lowicryl HM20 (Electron Microscopy Sciences, USA) was carried out with increasing concentrations of 10%, 25%, 50%, and 75%, for 2 h each. Infiltration with 10% and 25% was performed at −45 °C, and at 50% and 75% temperature was raised to −35 °C and −25 °C, respectively (5 °C/hour). Next, temperature was held at −25 °C for 30 h with 3 exchanges of 100% Lowicryl HM20 every 10 h, followed by polymerization of the resin under UV for additional 48 h at −25 °C. The temperature was then raised to 20 °C (5 °C/h), and further polymerization of the resin under UV for 12 h. In all, 500-nm-thick sections were cut using a Leica EM UC7 ultramicrotome (Leica Microsystems GmbH, Germany) mounted with a diamond knife (Diatome, Biel, Switzerland) and picked onto carbon-coated 200 mesh copper EM grids (Electron Microscopy Sciences, Hatfield, PA). 50 nm TetraSpeck microspheres (diluted 1:160 in PBS; Life Technologies), serving as fiducial markers for the correlation, were absorbed to the sections by placing the EM grids onto a 10 µl drop for 10 min, followed by three washes in DDW.

### Fluorescence microscopy of resin embedded samples

Imaging of EM grids by FM prior to electron tomography acquisition was performed as described in Kukulski et al.^47^. The EM grids were placed on a drop of water sandwiched between two glass coverslips (Menzel-Gläser, #1.5), sealed with grease, and imaged with the sections facing toward the objective in an Olympus IX83 fluorescence microscope controlled via VisiView software (Visitron Systems GmbH) and equipped with CoolLED pE-4000 light source (CoolLED Ltd., UK), an UAPON 100 × 1.49 NA oil immersion objective, and a Prime 95B sCMOS camera (Photometrics). Fluorescence excitation and emission were detected using filter-sets 470/40 nm and 525/50 nm for GFP, 560/40 nm and 630/75 nm for mCherry, and 620/60 nm and 700/75 nm MitoTracker Deep Red FM (Thermo Fisher Scientific).

### Electron tomography

Electron tomography (ET) was performed as previously described^48^ Tomographic fiducial markers (15 nm protein A-coupled gold beads) were adsorbed to both sides of the EM grids. Transmission electron microscopy (TEM) images for correlation, and Scanning transmission electron microscopy (STEM) tilt series were acquired using a Tecnai F20 (Thermo Fischer Scientific) at 200 kV, using SerialEM^49^. The TEM images for correlation were acquired at 4.47 nm pixel size using an UltraScan 4000 camera (Gatan). Tilt series were acquired over a 60° to −60° tilt range (1° increment) at 1.67 nm pixel size in nanoprobe mode using the bright-field detector, with a 10-µm C2 aperture and 320 mm camera length. The tomograms were reconstructed using IMOD software package (versions 4.9.4)^50^. Correlations of fluorescence and TEM images were performed using the ec-CLEM plugin with the Icy software^51^.

### Immunoprecipitation analysis for cellular protein interactions

HEK293 cells were seeded in 6-well plates and transiently transfected as described above. Eighteen hours post transfection cells were scraped and washed once in phosphate-buffered saline (PBS). Cell pellet was lysed for one hour at 4 °C in lysis buffer containing 50 mM Tris-Cl pH7.4, 150 mM NaCl, 0.5 mM EDTA, protease inhibitors and either 1% Lauryl Maltose Neopentyl Glycol (LMNG; Anatrace) or 0.5% Triton x-100. Lysate was then spun 10 min at 8000×*g*, 4 °C and pellet discarded. Lysate was incubated with 15 µl GFP-Trap® agarose beads (ChromoTek) for two hours in rotation at 4 °C. Agarose beads were then washed three times in buffer containing 50 mM Tris-Cl pH7.4, 150 mM NaCl and 0.1% LMNG or 0.1% Triton x-100. Protein was eluted from beads by incubating in 50 µl Laemmli sample buffer at 70 °C for 15 min. Samples from lysate (input) and eluate (pull-down) were loaded on either 4–20% mini-PROTEAN® (Bio-Rad) or 9% Laemmli gels^52^ and separated using SDS-PAGE. Proteins were detected using western blot. Briefly, proteins were transferred onto nitrocellulose membrane using the Trans-Blot®Turbo™ system (Bio-Rad), followed by blocking in SEA BLOCK buffer (Thermo Fisher Scientific). Membrane was then incubated with primary antibody overnight at 4 °C, washed three times in TBST, stained with secondary antibody, washed three times in TBST then once in PBS. Signal was detected using the LI-COR® Odyssey imaging system (LI-COR Biosciences). Primary antibodies used were anti-GFP (abcam, ab290), anti-Rab7A (Cell Signaling Technology, D95F2), anti-Protrudin (Proteintech 12680-1-AP), and anti-Actin (abcam, ab8224). Secondary antibodies used were IRDye^®^ 800CW Goat anti-Rabbit IgG and IRDye^®^ 800CW Goat anti-Mouse IgG (LI-COR Biosciences). Immunoprecipitation experiments from three biological replicates and representative blots are shown.

### Grazing incidence structured illumination microscopy

GI-SIM was performed in the previously described system^53^. Briefly, GI-SIM was built based on an inverted fluorescence microscope (IX83, Olympus).The light beams from a laser combiner equipped with 405 nm (LBX-405-300-CSB-PPA), 488 nm (Coherent, Genesis Max 488-500 STM), 560 nm (MPB Communications, VFL-P-500-560), and 642 nm (MPB Communications, 2RU-VFL-P-500-642) lasers were passed through an acousto-optic tunable filter (AOTF; AA Quanta Tech, AOTFnc-400.650-CPch-TN), which was used to dynamically adjust the excitation laser power coupled into backwards light path. The laser beam from AOTF was then expanded to a 1/e diameter of 15 mm and sent to a phase-only modulator consisting of a polarizing beam splitter, a achromatic half-wave plate (HWP; Bolder Vision Optik, BVO AHWP3), and a ferroelectric spatial light modulator (SLM; Forth Dimension Displays, SXGA-3DM). The light diffracted by the grating pattern displayed on SLM passed through a polarization rotator consisting of three pairs of segmented HWP, which rotates the linear polarization of the diffracted light so as to maintain the S-polarization necessary to maximize the pattern contrast for all pattern orientations. The fluorescent image generated by the applied excitation pattern of each phase and orientation was collected by a high numerical aperture objective (Olympus APON 100XHOTIRF 1.7 NA), and then separated by a dichroic beam splitter (Chroma, ZT405/488/560/647tpc), and focused by a tube lens onto a sCMOS camera (Hamamatsu, Orca Flash 4.0 v3). The nine raw images consisting of 3-orientation and 3-phase for each time point were reconstructed into a super-resolution (SR) image. COS-7 cells were plated on collagen coated high-NA coverslips and transfected on the following day. The typical acquisition speed for each multi-color GI-SIM super-resolution imaging were 1 frame/s under 50 W/cm^2^ excitation intensity. Cells were imaged in a microscope stage top micro incubator to maintain the 37 °C and 5% CO_2_. When cells were transfected with Snap-OMM plasmids, the specimen was labeled with 647-SiR substrate (S9102S, NEB) for 20 min, and then imaged immediately.

### Statistics and reproducibility

Immunoprecipitation/mass spectrometry experiments were performed in three different human cell lines: HCT116, HEK293, and human fibroblasts, each with three biological replicates. All remaining immunoprecipitation experiments were from three biological replicates and representative blots are shown. Live imaging of transiently transfected cells expressing full length PDZD8-GFP/PDZD8-mCherry was performed in multiple cell lines: U2OS, COS7 and HeLa. Cells expressing PDZD8-GFP/mCherry were imaged in over 70 independent live-cell imaging sessions, cells expressing Protrudin-GFP/mCherry were imaged in 55 different sessions and cells expressing different PDZD8 truncated constructs were each imaged between 4 and 25 different sessions. GI-SIM imaging of cells was conducted from 2 to 8 times in independent live imaging sessions. Images from indirect immunofluorescence of native PDZD8 using anti-PDZD8 antibodies in cells expressing Rab7-GFP represent are representative from four biological replicates. In all cases, the number of cell analyzed for each imaging condition was greater than *n* = 100.

### Reporting summary

Further information on research design is available in the Nature Research Reporting Summary linked to this article.

## Supplementary information

Supplementary Information

Supplementary Dataset 1

Supplementary Dataset 2

Supplementary Movie 1

Supplementary Movie 2

Supplementary Movie 3

Supplementary Movie 4

Reporting Summary

## Data Availability

Mass spectrometry analysis results for the interactomes of PDZD8 and Protrudin are provided with the manuscript as Supplementary Dataset 1 and Supplementary Dataset 2. The proteomic datasets that support these analyses are available in the PRoteomics IDEntifications (PRIDE) database via ProteomeXchange with identifier PXD015523 [http://www.ebi.ac.uk/pride/archive/projects/PXD015523]. Source data are provided with this paper. Other data are available from the corresponding author upon reasonable request.

## Source data

Source Data
