# Supplementary material for: PDZD8 interacts with Protrudin and Rab7 at ER-late endosome membrane contact sites associated with mitochondria
Source: Nat Commun. 2020 Jul 20;11:3645. doi: 10.1038/s41467-020-17451-7 (PMC7371716; doi:10.1038/s41467-020-17451-7)
Supplement: Supplementary file 1 — Supplementary Information [file 41467_2020_17451_MOESM1_ESM.pdf]

PDZD8 interacts with Protrudin and Rab7 at ER-late endosome membrane contact sites that associate with mitochondria

Elbaz-Alon et al.

Supplementary information includes:

Supplementary Figures 1-7
Supplementary Table 1
Supplementary Table 2

Supplementary Figure 1

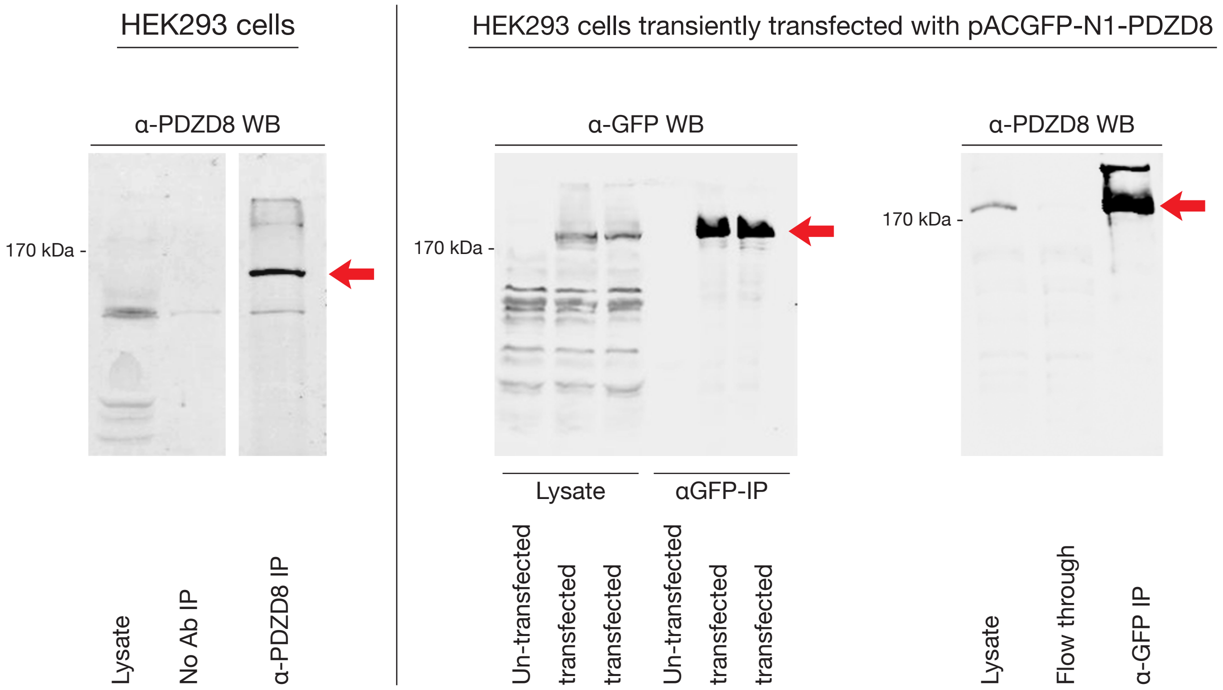

Lysate loaded (µg total protein)	48	40	60
IP (mg total protein)	0.8	0.5	0.75

Endogenous PDZD8 and transiently transfected PDZD8-GFP levels.

Extracts from HEK293T cells (left panel) and HEK293 cells transiently transfected with pACGFP-N1-PDZD8-GFP were immunoprecipitated with an anti-PDZD8 antibody attached to protein G beads (left panel) or anti-GFP-Trap beads (middle and right panels) and Western blot analysis was performed using anti-PDZD8 (left and right panels) or anti-GFP (middle panel) antibodies. Red arrow indicates PDZD8 on blot. Source data are provided as a source data file.

Supplementary Figure 2

A

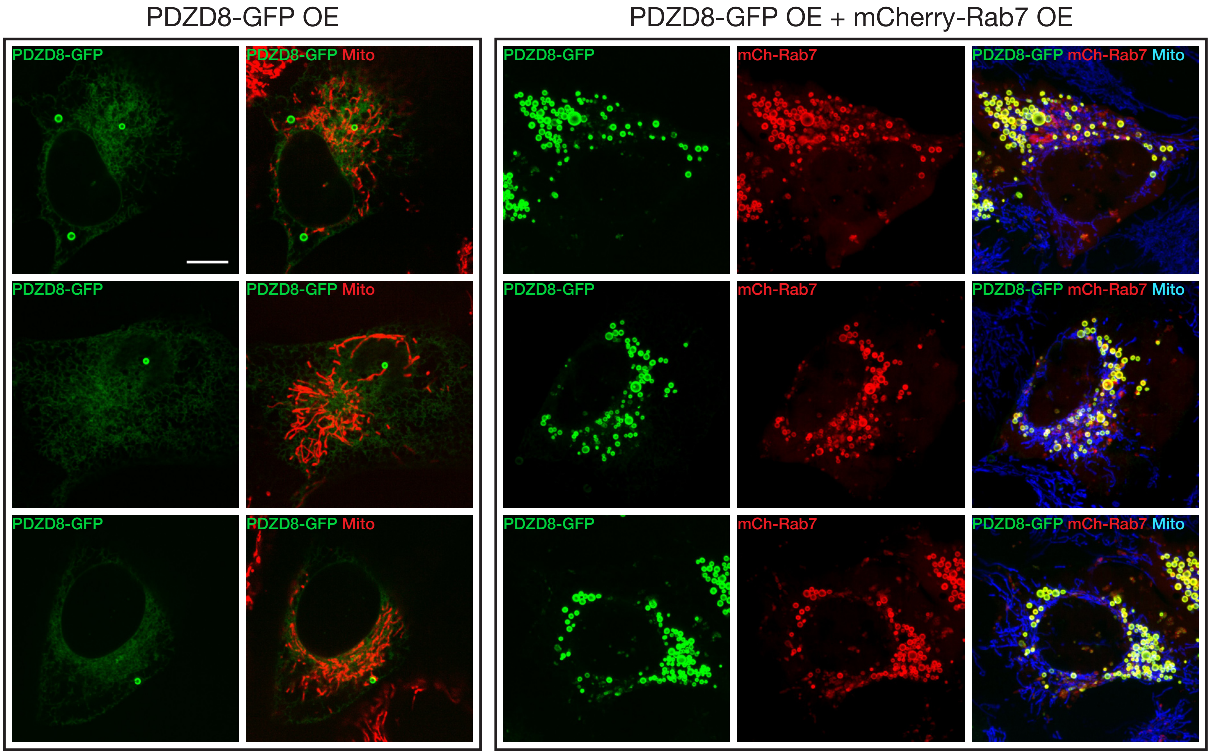

B

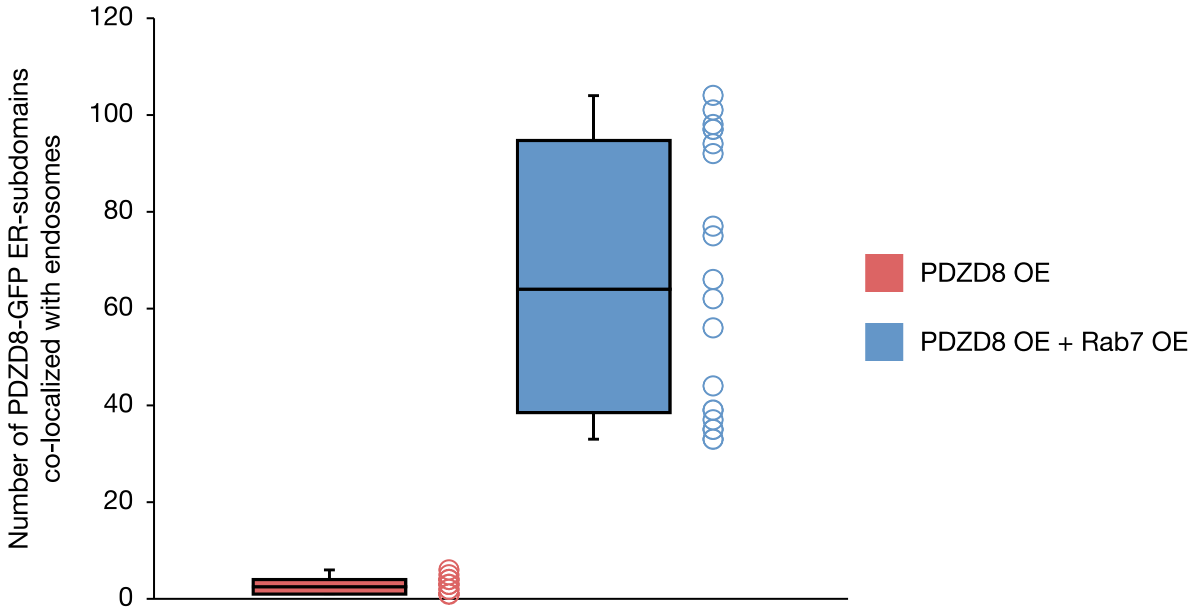

PDZD8/Rab7 overexpression increases late endosome-associated PDZD8 subdomains.

(**A**) Representative images of U2OS cells expressing PDZD8-GFP (green) versus cells co-expressing PDZD8-GFP (in green) and mCherry-Rab7 (in red). The number of PDZD8 mediated ER-LE contacts in each cell was inferred from spherical PDZD8-GFP labeled regions of relative high fluorescence intensity. In cells where PDZD8-GFP and mCherry-Rab7 were overexpressed together areas of co-localization between PDZD8 and Rab7 were counted as ER-late endosome MCSs. Mitochondria (in blue) labeled with MitoTracker DeepRed. Scale bar: 10µm. Images are single planes. (**B**) Data from n=20 cells is summarized in a box-and-whisker plot. For PDZD8-GFP alone min=1, median=2.5, max=6, bounds of box: Q1=1, Q3 =4. For PDZD8-GFP co-expressed with mCherry-Rab7 min=33, median=64, max=104, bounds of box: Q1=38.5 ,Q3=94.75. The average number of PDZD8-GFP labeled subdomains was 2.5±1.5 per cell compared to 65.7±27.4 per cell when co-expressed with mCherry-Rab7 (mean ± standard deviation). Source data are provided as a source data file.

Supplementary Figure 3

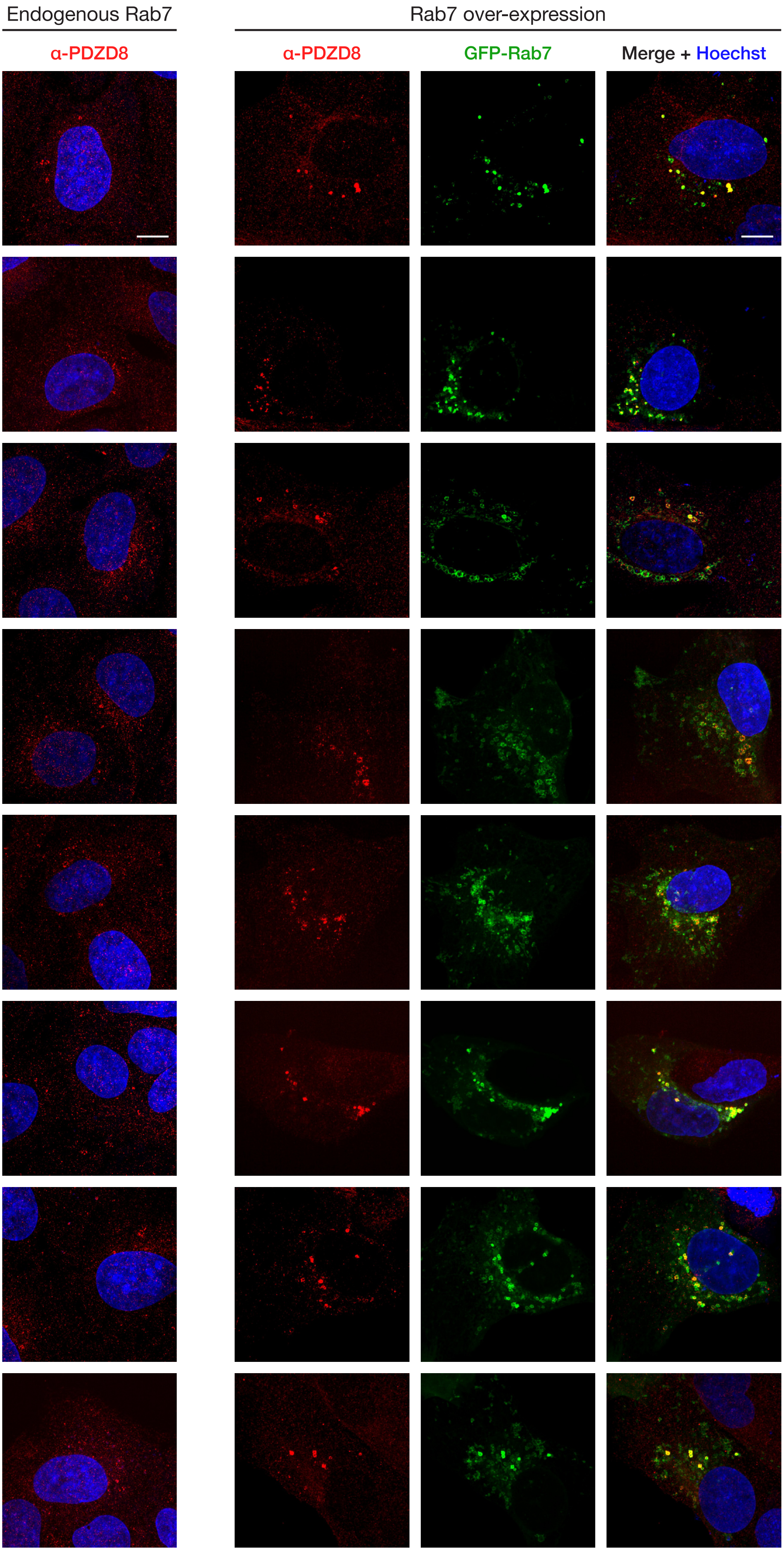

Endogenously expressed PDZD8 is recruited to Rab7-labeled late endosomes.

Immunofluorescence analysis of native U2OS cells (left panel) versus U2OS cells expressing GFP-Rab7 (in green; right panel). Cells were fixed, incubated with an anti-PDZD8 (in red) antibody followed by a secondary anti-rabbit AlexaFluor568 antibody and stained with Hoechst stain (labeling the nucleus; in blue). Scale bar: 10μm.

Supplementary Figure 4

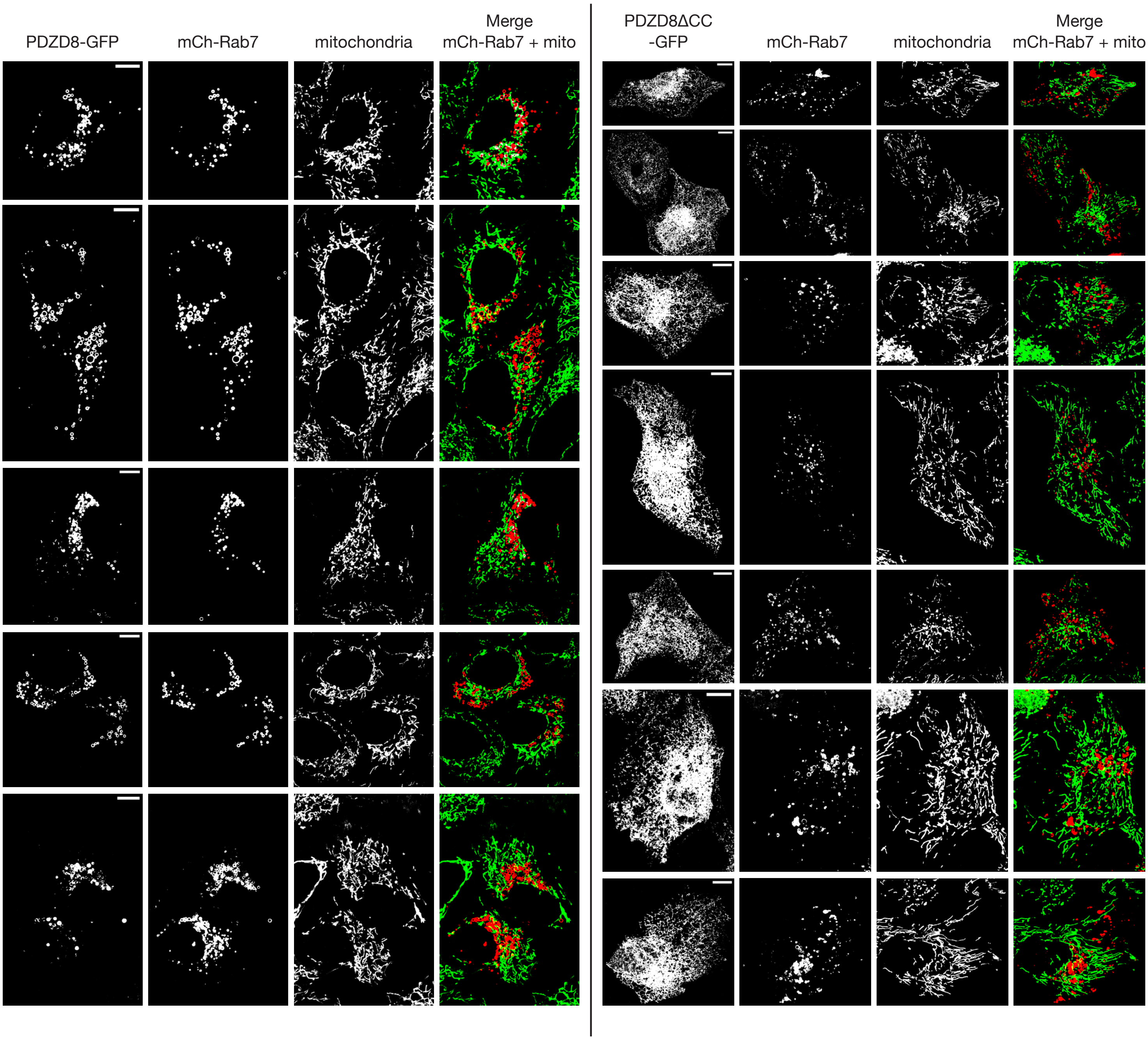

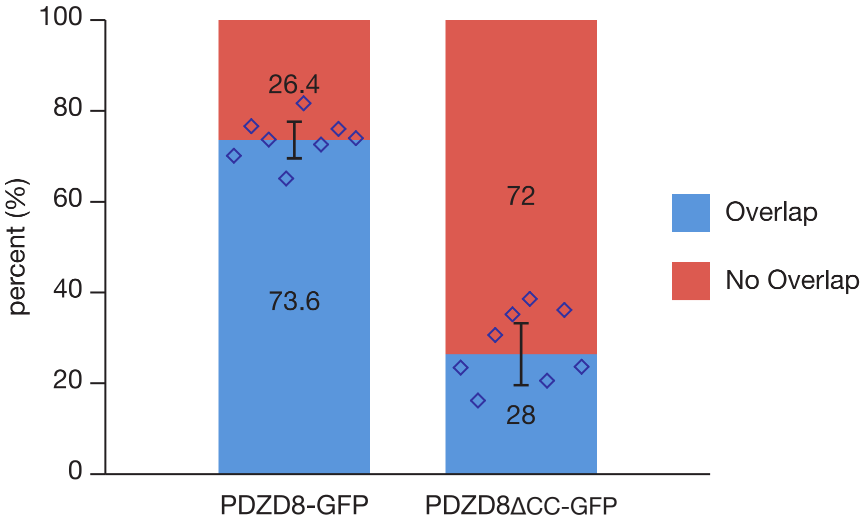

Overexpression of PDZD8 enhances mitochondrial association with late endosomes in cells.

Single plane images of U2OS cells overexpressing mCherry-Rab7 and either PDZD8-GFP or PDZD8ΔCC-GFP were analyzed by thresholding (ImageJ) and then comparing the number of mCherry-labeled late endosomes whose signal co-localized with MitotrackerDeepRed-labeled mitochondria out of the total number of late endosomes in the cell. The ratio was calculated for each cell and presented as percent (%). Results for n=8 cells for each condition (total of 564 late endosomes counted in cells expressing PDZD8-GFP and mCherry-Rab7, and 706 late endosomes counted in cells expressing PDZD8ΔCC-GFP and mCherry-Rab7) are presented in a bar plot as mean ± SD. Source .data are provided as a source data file. Scale bar: 10µm

Supplementary Figure 5

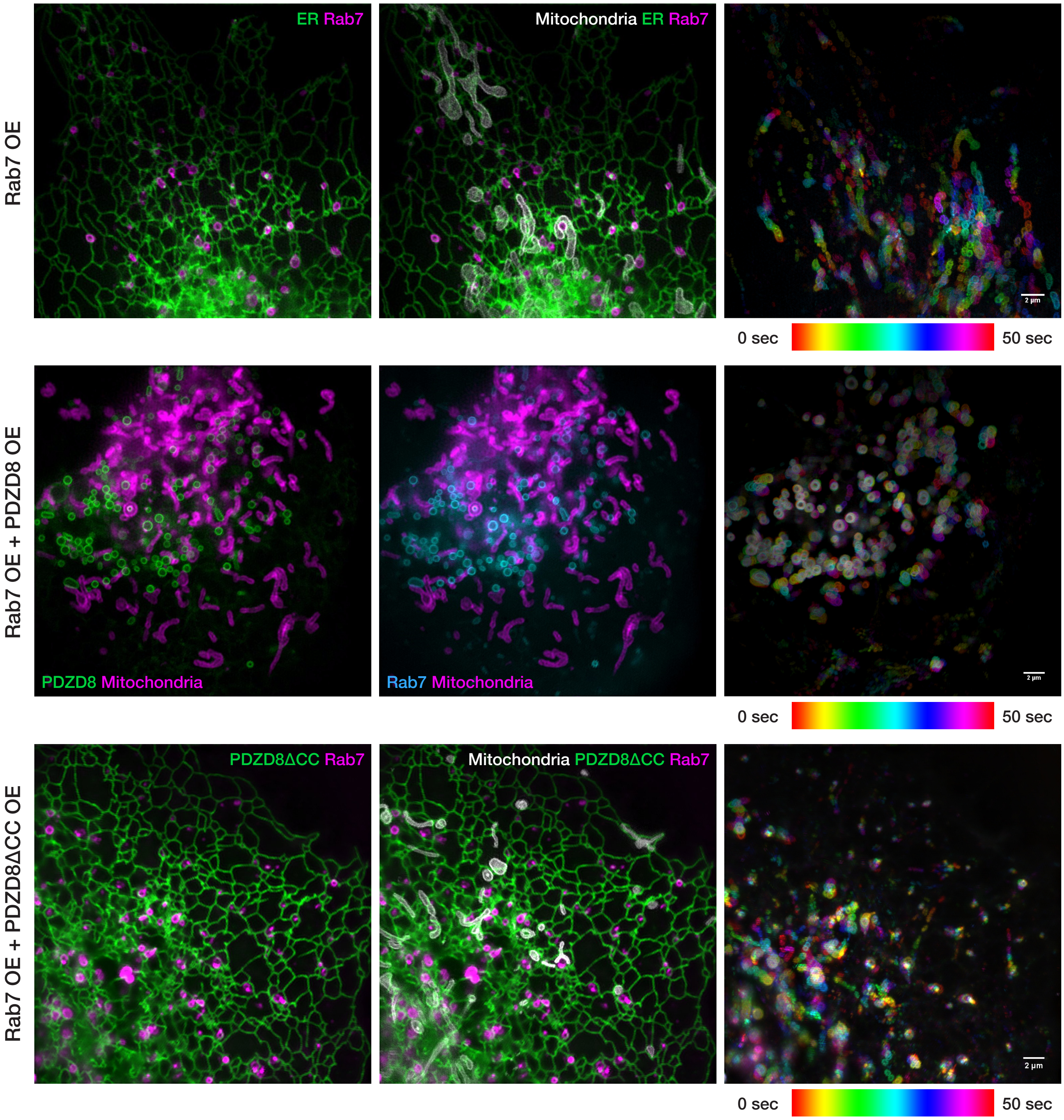

Mitochondria co-localize with ER-LE contacts marked by PDZD8-GFP and BFP-Rab7.

Late endosomal motility is impaired in cells overexpressing PDZD8. Representative images from time lapse GI-SIM of COS7 cells expressing BFPRab7with either a general Emerald-Sec61β-ER marker (upper panel; Corresponding supplementary movie file M1), PDZD8-GFP (middle panel; Corresponding supplementary movie file M2) or with PDZD8ΔCC-GFP (lower panel, Corresponding supplementary movie file M3), analyzed as temporal color-coded representations. Each GI-SIM image of late endosome is color coded according to its acquisition time point over 50 sec interval, and then a total 50 color-coded images were projected to form a single image, demonstrating the dynamics of Rab7-labeled late endosomes. White = static late endosomes and multiple colors = motile late endosomes. Scale bar: 2μm. Mitochondria labeled with Snap-OMM (mitochondrial outer membrane marker).

Supplementary Figure 6

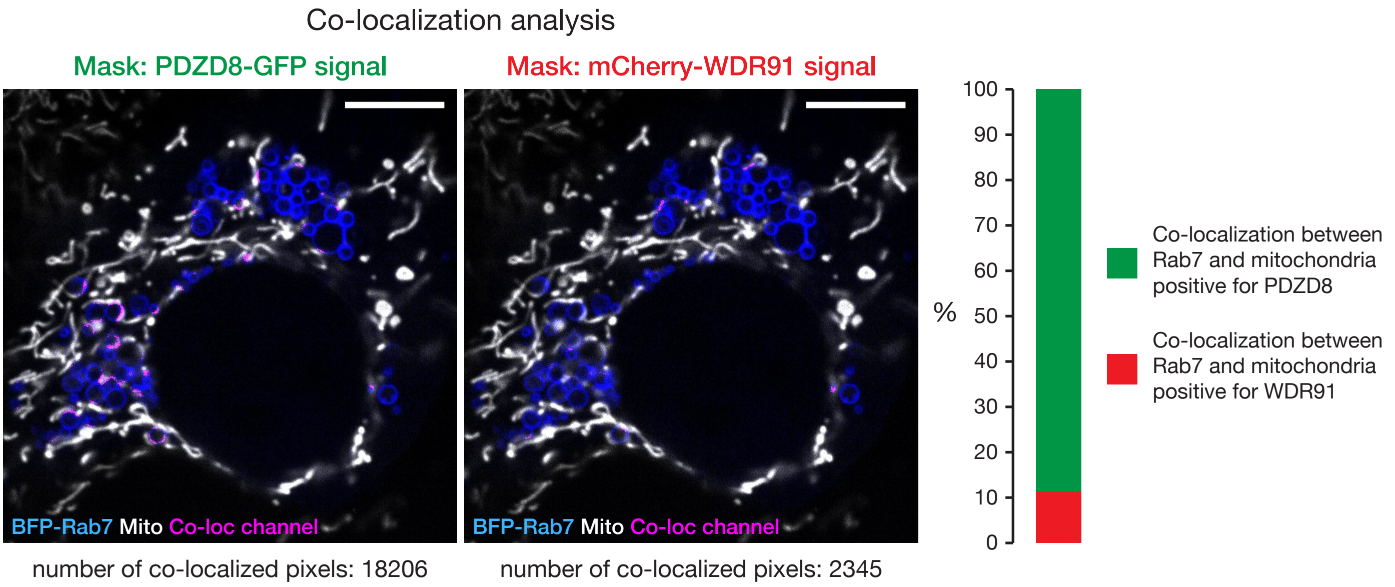

Mitochondria co-localize with PDZD8 at Rab7-labeled endosomes.

Analysis of the amount of contact area (in pixels) between BFP-Rab7-labeled late endosomes (in blue) and mitochondria (in white); a comparison of PDZD8-GFP labeled area versus mCherry-WDR91 labeled area was performed using the software Imaris. A stack of 17 images (z-planes of a single cell) were analyzed using the IMARIS software ImarisCoLoc module. As PDZD8 and WDR91 both interact and co-localize with Rab7 on late endosomes in a mutual exclusive manner, we used the Mask channel feature to define either the PDZD8-GFP or the mCherry-WDR91 as a masking area for the entire analysis. We used that to calculate the relative area of late-endosome (inferred from BFP-Rab7 labeling) contact with MitoTracker-labeled mitochondria that each of these proteins co-localizes with. We then used the Build coloc channel feature in order to save the result of colocalization for each analysis as a separate channel that can be viewed (in purple) as an image for each single plane analyzed. The total contact area (in pixels) through all 17 planes between the BFP-Rab7 signal (late endosomes) and Mitotracker labeled mitochondria was calculated through a PDZD8-GFP masked signal (left image) and then through a mCherry-WDR91 masked based signal (right image) to create a co-localization image for each. The relative area that PDZD8 and WDR91 occupied on the total late-endosome-mitochondria interface is represented in a bar plot. Scale bar: 10μm.

Supplementary Figure 7

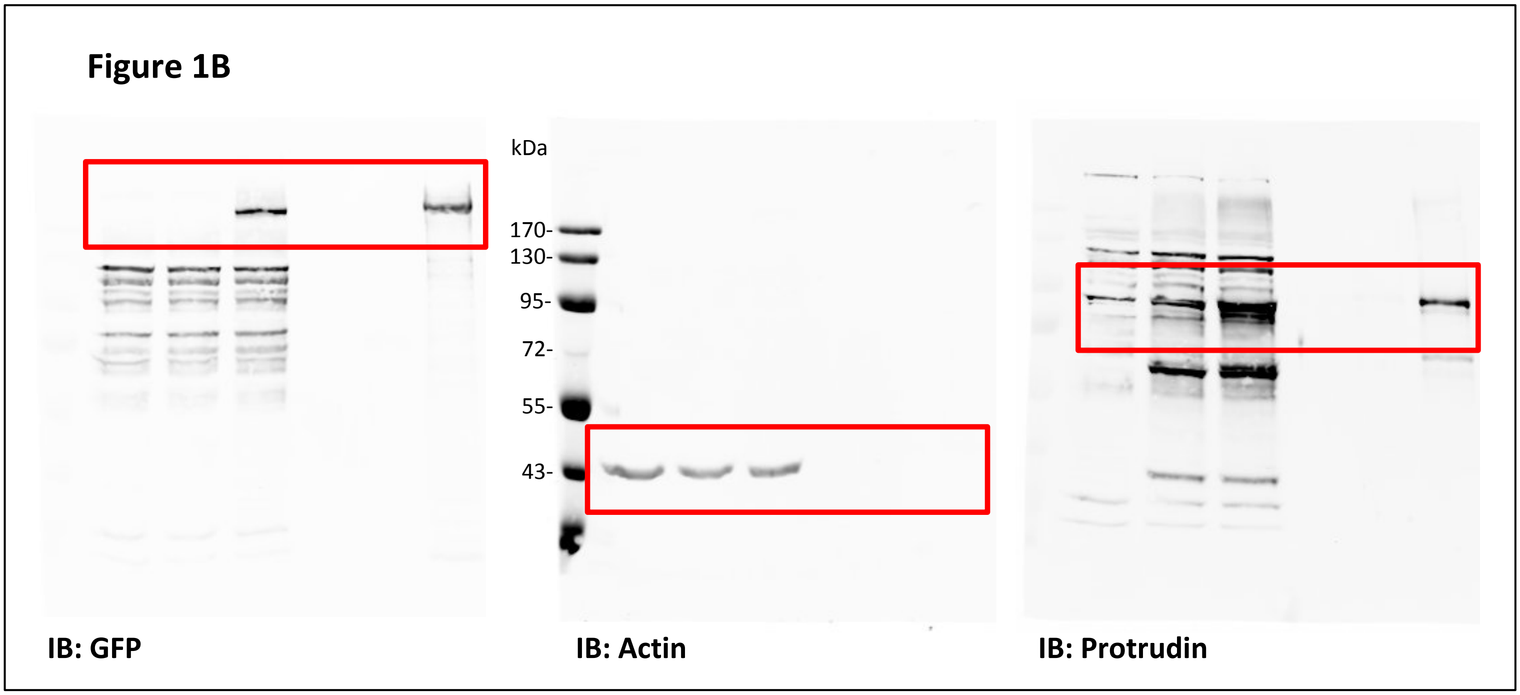

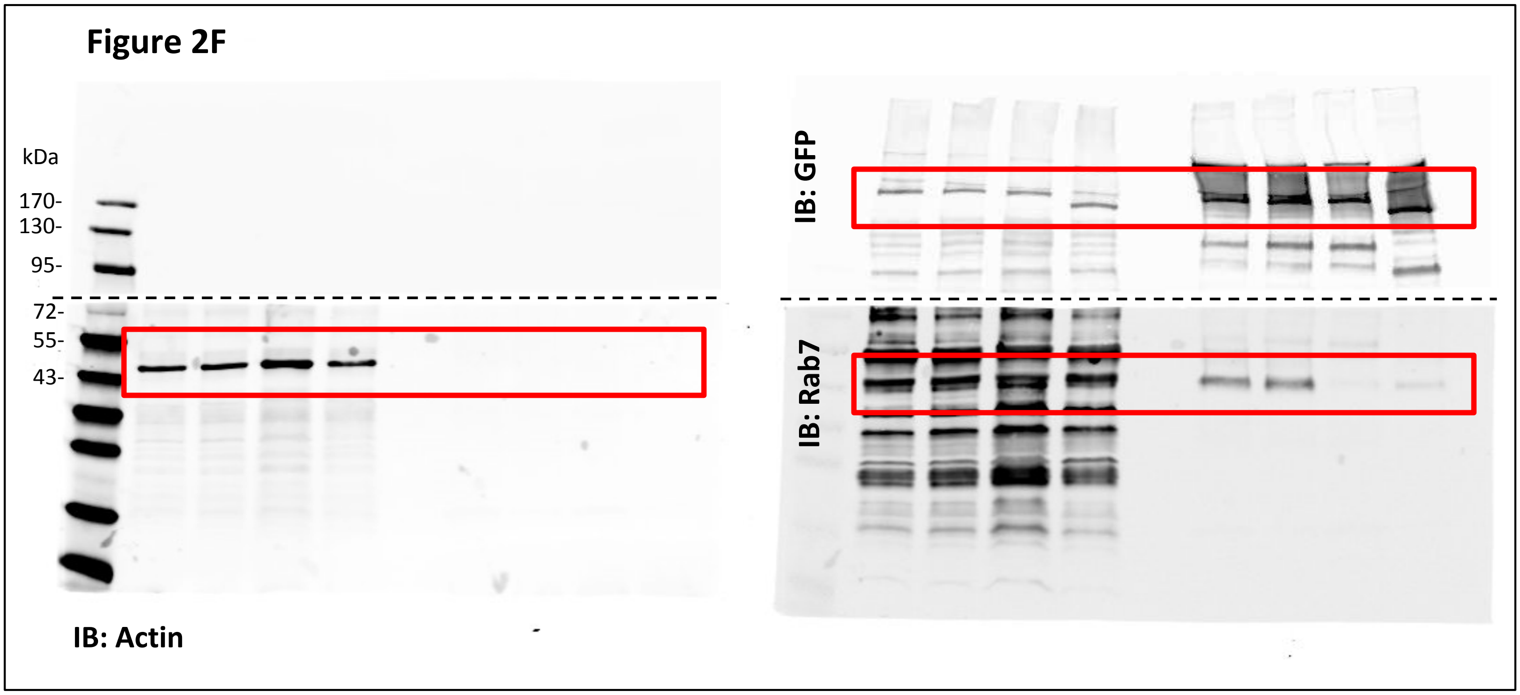

Figure 4B

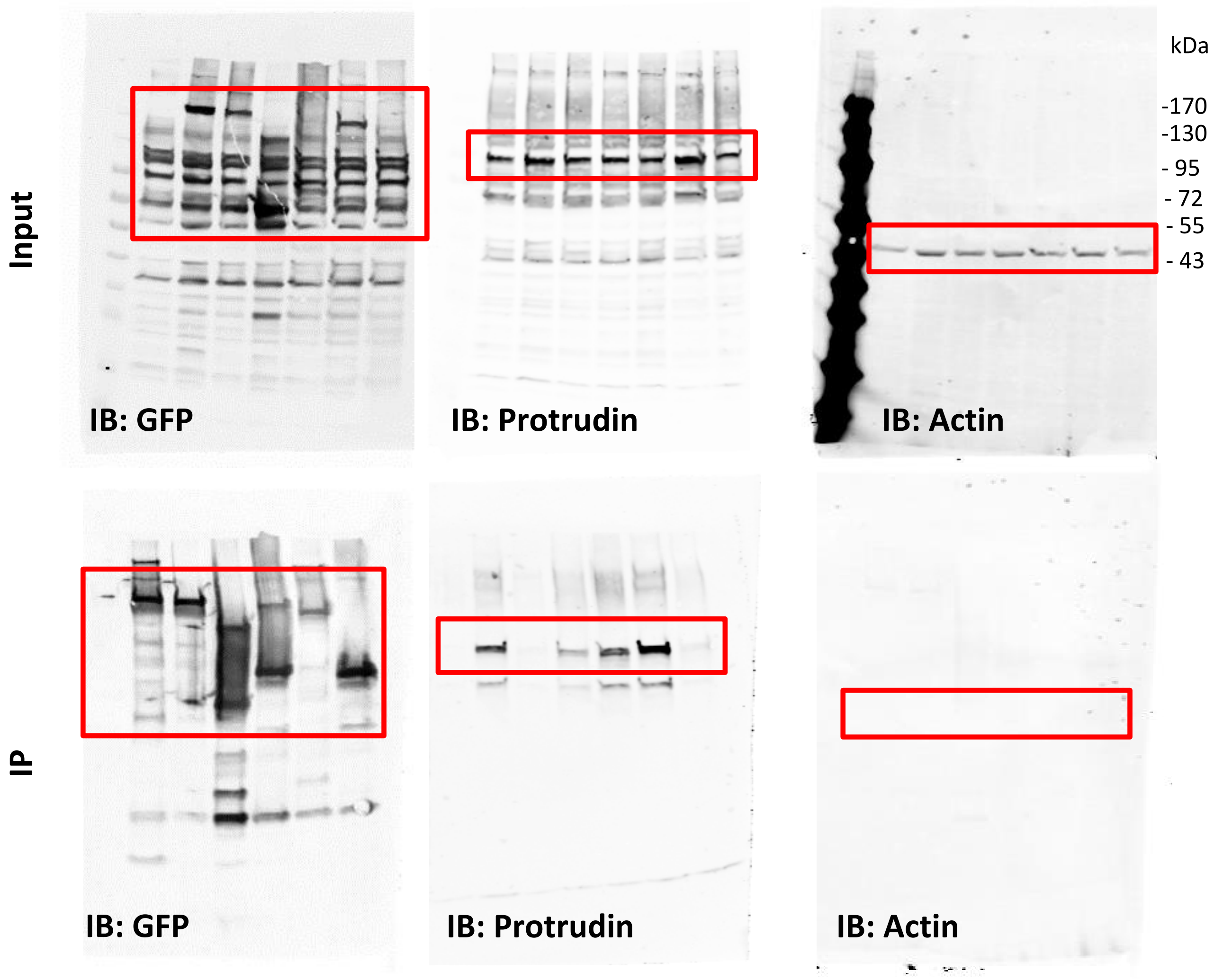

Input
IP
IB: GFP
IB: Protrudin
IB: Actin
IB: GFP
IB: Protrudin
IB: Actin
kDa
-170
-130
- 95
- 72
- 55
- 43

Supplementary Figure 1

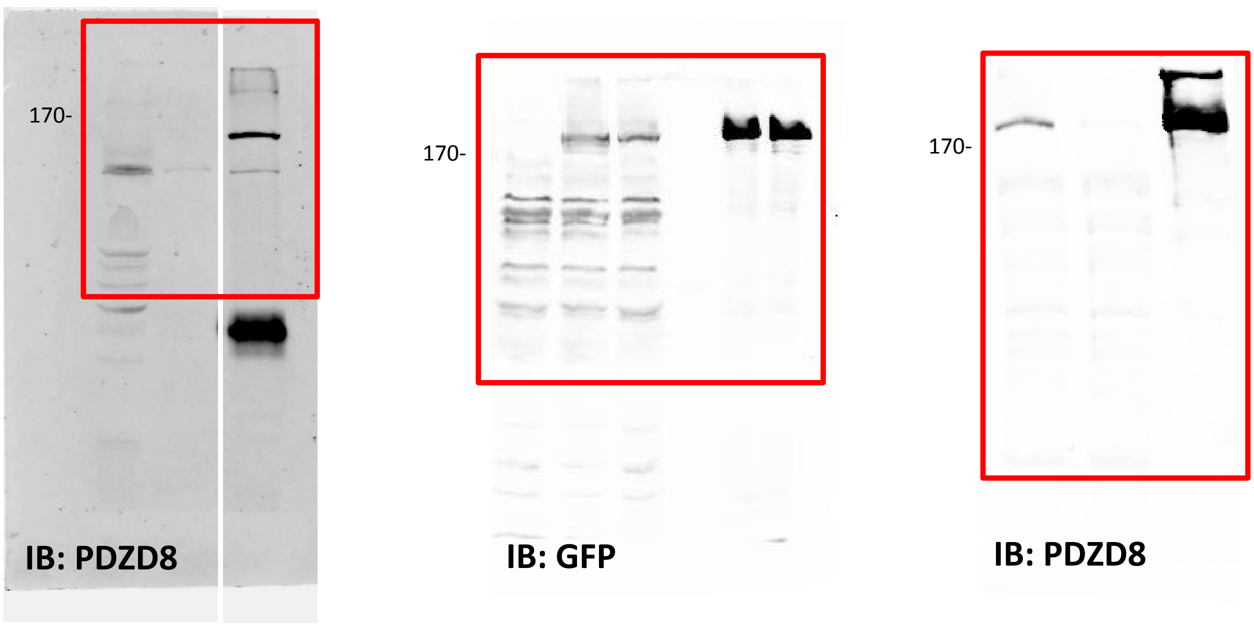

Uncropped images for scanned Western blots contained in Figures 1B, 2F and 4B and Supplementary Figure 1.

.Source data are provided as a Source Data file

Supplementary table 1: Plasmids used in this study

Construct	Plasmid	Source
PDZD8-GFP (Full length)	pACGFP-N1	This study
PDZD8 (aa27-1154)-GFP	pACGFP-N1	This study
PDZD8 (aa1-1000)-GFP	pACGFP-N1	This study
PDZD8 (aa1-470)-GFP	pACGFP-N1	This study
PDZD8 (aa1-300)-GFP	pACGFP-N1	This study
PDZD8 (aa27-470)-GFP	pACGFP-N1	This study
PDZD8 (aa90-300)-GFP	pACGFP-N1	This study
PDZD8 (aa300-1154)-GFP	pACGFP-N1	This study
PDZD8 (aa800-1154)-GFP	pACGFP-N1	This study
PDZD8 (aa1-1000)-mCherry	pACGFP-N1	This study
mCherry-Rab7a		Addgene # 61804
mCherry-Rab5		Addgene # 49201
Lamp1-mCherry	pAC-mCherry-N1	Kind gift from Katherine Labbe, UC Davis
BFP-SEC61beta		Addgene # 49154
GFP-VAPA	pACGFP-C1	This Study
mCherry-Rab7a-T22N	pAC-mCherry-C1	This Study
mCherry-Rab7a-Q67L	pAC-mCherry-C1	This Study
BFP-Rab7a	pAC-EBFP-C1	This Study
mCherry-WDR91	pAC-mCherry-C1	This Study
PDZD8-Cherry	pAC-mCherry-N1	This Study
Protrudin-GFP	pACGFP-N1	This study

Supplementary table 2: DNA primers list

**Plasmid**	**Primer sequence 5’-3’**	**Remarks**
pACGFP-N1-PDZD8 / pACmCH-N1-PDZD8	Forward - TTATTATGAATTCATGGGGCTGCTGCTCATGATCCTGGCG<br>Reverse - TATAATTCCCGGGCCACAGACTCGGATGGGCCAAATAAG	Cloning full length PDZD8 into pAC-N1 plasmids using EcoRI/XmaI restriction sites
pACGFP-N1-Protrudin / pACmCH-N1-Protrudin / pACGFP-N1-ProtrudinFYVE4A	Forward - TTATTATGAATTCATGCAGACATCAGAACGTGAGGGGAGTGGG<br>Reverse - TATAATTCCCGGGCCTTGCTCAAGGTCTGGTTACACGAGGC	Cloning full length Protrudin and Protrudin FYVE4A mutant into pAC-N1 plasmids using EcoRI/XmaI restriction sites
pACGFP-N1-PDZD8 27-1154 / pACmCH-N1-PDZD8 27-1154	Forward -TTATTATGAATTCATGTACCGCAGACAGCCCGAGCCGCCGGC<br>Reverse - TATAATTCCCGGGCCACAGACTCGGATGGGCCAAATAAG	
pACGFP-N1-PDZD8 27-470	Forward -TTATTATGAATTCATGTACCGCAGACAGCCCGAGCCGCCGGC<br>Reverse - TATAATTCCCGGGGCAAAAAGTTTTCTTCCAACTGGCCAAAGTT	Cloning truncated versions of PDZD8 into pAC-N1 plasmids using EcoRI/XmaI restriction sites
pACGFP-N1-PDZD8 90-300	Forward -TTATTATGAATTCATGACGCGGGAGACTTGCTACTTCCTC<br>Reverse -TATAATTCCCGGGCGGTCTGGTATGGAAAAAACGGCTTAAAC	
pACGFP-N1-PDZD8 1-300	Forward - TTATTATGAATTCATGGGGCTGCTGCTCATGATCCTGGCG<br>Reverse -TATAATTCCCGGGCGGTCTGGTATGGAAAAAACGGCTTAAAC	
pACGFP-N1-PDZD8 1-470	Forward - TTATTATGAATTCATGGGGCTGCTGCTCATGATCCTGGCG<br>Reverse -TATAATTCCCGGGGCAAAAAGTTTTCTTCCAACTGGCCAAAGTT	
pACGFP-N1-PDZD8 1-1000	Forward - TTATTATGAATTCATGGGGCTGCTGCTCATGATCCTGGCG<br>Reverse -TATAATTCCCGGGCCTTTATTCCTGTGCTGTTTCC	
pACGFP-N1-PDZD8 300-1154	Forward -TTATTATGAATTCATGTTGCAAGGATTTGAAGAAGATGAAG<br>Reverse - TATAATTCCCGGGCCACAGACTCGGATGGGCCAAATAAG	
pACGFP-N1-PDZD8 800-1154	Forward -TTATTATGAATTCATGTCAGACCACCATGTAGTTAC<br>Reverse - TATAATTCCCGGGCCACAGACTCGGATGGGCCAAATAAG	
pACGFP/EBFP/mCH-C1-Rab7	Forward - ATATATTTAGAATTCTATGACCTCTAGGAAGAAAGTG<br>Reverse - ATATATTAACCCGGGTCAGCAACTGCAGCTTTCTGCCG	Cloning of wt RAB7A, RAB7A (T22N) and RAB77A (Q67L) into pAC-C1 plasmids using EcoRI/XmaI restriction sites
pACmCH-C1-WDR91	Forward - TTATTATGAATTCATGGCGGAGGCCGTGGAGCGCAC<br>Reverse - TATAATTCCCGGGCGGCTTTATGGGCCAGGAGGGTGGTCAG	Cloning of WDR91 into pAC-C1 plasmid using EcoRI/XmaI restriction sites
pACGFP-C1 VAPA	Forward -ATATATTTAGAATTCTATGGCGTCCGCCTCAGGGGCCATGGCGAAG<br>Reverse -ATATATTAACCCGGGCTACAAGATGAATTTCCCTAGAAAG	Cloning of VAPA into pAC-C1 plasmid using EcoRI/XmaI restriction sites
