# Supplementary material for: PDZD8 interacts with Protrudin and Rab7 at ER-late endosome membrane contact sites associated with mitochondria
Source: Nat Commun. 2020 Jul 20;11:3645. doi: 10.1038/s41467-020-17451-7 (PMC7371716; doi:10.1038/s41467-020-17451-7)
Supplement: Supplementary file 2 — Supplementary Dataset 1 [file 41467_2020_17451_MOESM2_ESM.pdf]

Supplementary Data File 1: Potential interactors of PDZD8. Mass-spectrometry based results from an analysis of immunoprecipitates using an anti-PDZD8 antibody from crosslinked human HCT116 cell lysates. Significant potential interactors were identified using one-sided Student's t-test (FDR 0.05, S0=0.1)

Gene names	Protein names	Student's T-test Significant Control_PDZD8	-Log Student's T-test p-value Control_PDZD8	Student's T-test q-value Control_PDZD8	Student's T-test Difference Control_PDZD8
PDZD8	PDZ domain-containing protein 8	+	4.66815	0	-11.1716
ZFYVE27	Protrudin	+	5.33844	0	-8.58987
RAB7A	Ras-related protein Rab-7a	+	2.3438	0.0440513	-1.30709
OSBP	Oxysterol-binding protein 1	+	2.58845	0.0293333	-3.48095
OSBPL8	Oxysterol-binding protein-related protein 8;Oxysterol-bindin	+	4.18329	0.0213333	-4.03666
OSBPL3	Oxysterol-binding protein-related protein 3	+	5.11757	0	-3.95279
VAPB	Vesicle-associated membrane protein-associated protein B/(	+	3.19562	0.03125	-3.57987
VAPA	Vesicle-associated membrane protein-associated protein A	+	2.92095	0.0260571	-2.34448
TRIM21	E3 ubiquitin-protein ligase TRIM21	+	2.98265	0.025	-7.56452
IGHG1;IGHG3	Ig gamma-1 chain C region;Ig gamma-3 chain C region	+	2.72657	0.0285	-6.20405
PNPLA6	Neuropathy target esterase	+	4.3001	0	-4.92869
RINT1	RAD50-interacting protein 1	+	2.28078	0.04	-4.23737
PPFIA1	Liprin-alpha-1	+	2.8897	0.0282667	-4.11933
STX10	Syntaxin-10	+	3.32647	0.0317143	-3.75113
RAB3GAP1	Rab3 GTPase-activating protein catalytic subunit	+	4.22676	0.016	-3.52278
DNM1L	Dynamin-1-like protein	+	4.01617	0.0182857	-5.34403
STIM1	Stromal interaction molecule 1	+	3.5755	0.0324	-3.45548
FAT1	Protocadherin Fat 1;Protocadherin Fat 1, nuclear form	+	3.50109	0.031	-3.38028
TACC1	Transforming acidic coiled-coil-containing protein 1	+	3.55419	0.0338182	-3.09895
NFXL1	NF-X1-type zinc finger protein NFXL1	+	2.64322	0.0295814	-3.02774
ANKLE2	Ankyrin repeat and LEM domain-containing protein 2	+	3.25989	0.0277778	-2.95373
NBAS	Neuroblastoma-amplified sequence	+	2.11587	0.0440556	-2.92929
UBXN4	UBX domain-containing protein 4	+	2.09401	0.0458133	-2.91631
CEP170	Centrosomal protein of 170 kDa	+	3.72808	0.0257778	-2.90229
EMC2	ER membrane protein complex subunit 2	+	4.94348	0	-2.88812
STIM2	Stromal interaction molecule 2	+	3.02718	0.0273103	-2.88682

WFS1	Wolframin	+	2.71317	0.0272	-2.88342
ESYT1	Extended synaptotagmin-1	+	3.04807	0.026963	-2.87975
JAKMIP1	Janus kinase and microtubule-interacting protein 1	+	2.19515	0.0427879	-2.74533
SRPR	Signal recognition particle receptor subunit alpha	+	2.70045	0.029561	-2.69471
TRIM4	E3 ubiquitin-protein ligase TRIM4	+	2.9473	0.0276364	-2.65648
EIF4E	Eukaryotic translation initiation factor 4E	+	2.94981	0.0268235	-2.63271
SDF2L1	Stromal cell-derived factor 2-like protein 1	+	2.2022	0.0421493	-2.63182
CD2AP	CD2-associated protein	+	3.23403	0.0254545	-2.54724
DNAJB12	DnaJ homolog subfamily B member 12	+	2.08081	0.0449383	-2.54073
CALU	Calumenin	+	2.67351	0.0280851	-2.5065
MVD	Diphosphomevalonate decarboxylase	+	3.35521	0.0263158	-2.45525
HOOK2	Protein Hook homolog 2	+	2.72965	0.0288571	-2.44599
CNOT3	CCR4-NOT transcription complex subunit 3	+	2.25168	0.0428571	-2.40758
KTN1	Kinectin	+	2.88535	0.0273333	-2.38926
MFF	Mitochondrial fission factor	+	2.61889	0.0265946	-5.61441
OS9	Protein OS-9	+	3.20596	0.0264348	-2.33754
SCFD1	Sec1 family domain-containing protein 1	+	2.74428	0.0289091	-2.28463
COPA	Coatomer subunit alpha;Xenin;Proxenin	+	3.46767	0.0333333	-2.25454
STX18	Syntaxin-18	+	2.49829	0.0348148	-2.23959
CDKAL1	Threonylcarbamoyladenosine tRNA methylthiotransferase	+	3.1628	0.0282857	-2.20484
KIF21A	Kinesin-like protein KIF21A	+	2.19592	0.043831	-2.19906
SRP19	Signal recognition particle 19 kDa protein	+	3.24183	0.0253333	-2.18666
INF2	Inverted formin-2	+	2.42881	0.0413793	-2.12068
SREBF1	Sterol regulatory element-binding protein 1;Processed sterol	+	3.1259	0.0294194	-2.0837
PRKAR1A	cAMP-dependent protein kinase type I-alpha regulatory subu	+	3.49905	0.0294118	-2.04123
MVP	Major vault protein	+	3.83888	0.0341538	-1.97817
PLOD1	Procollagen-lysine,2-oxoglutarate 5-dioxygenase 1	+	2.32848	0.0421875	-1.86901
ATF6	Cyclic AMP-dependent transcription factor ATF-6 alpha;Proc	+	2.37948	0.0407097	-1.85956
PSMD5	26S proteasome non-ATPase regulatory subunit 5	+	2.67715	0.0336	-1.85692
PTBP3	Polypyrimidine tract-binding protein 3	+	2.60893	0.0336923	-1.83445
UFL1	E3 UFM1-protein ligase 1	+	3.36653	0.02672	-1.79136
ESYT2	Extended synaptotagmin-2	+	2.33583	0.0415385	-1.75636
SEC22B	Vesicle-trafficking protein SEC22b	+	2.50504	0.040678	-1.74633

VPS26B	Vacuolar protein sorting-associated protein 26B	+	2.49904	0.04	-1.73288
ITGB4	Integrin beta-4	+	2.7883	0.0311667	-1.64648
HLA-C	HLA class I histocompatibility antigen, Cw-6 alpha chain;HLA	+	2.17397	0.0497073	-1.62657
PTPN1	Tyrosine-protein phosphatase non-receptor type 1;Tyrosine-	+	2.3789	0.0415294	-1.51244
SRP54	Signal recognition particle 54 kDa protein	+	2.54855	0.0405246	-1.48667
LIMD1	LIM domain-containing protein 1	+	2.31273	0.0455676	-1.4699
BRAT1	BRCA1-associated ATM activator 1	+	2.8588	0.0318367	-1.45555
SPATA5	Spermatogenesis-associated protein 5	+	2.98225	0.0286957	-1.44132
PRDX4	Peroxiredoxin-4	+	3.58494	0.028	-1.40399
ERLIN1	Erlin-1	+	3.20754	0.0258947	-1.39008
BNIP1	Vesicle transport protein SEC20	+	2.41455	0.0417971	-1.38768
CSK	Tyrosine-protein kinase CSK	+	2.79964	0.0354717	-1.30234
EMD	Emerin	+	2.78086	0.0375714	-1.24557
SRP68	Signal recognition particle subunit SRP68	+	2.36214	0.0434937	-1.24397
SPTLC1	Serine palmitoyltransferase 1	+	2.39035	0.0452105	-1.23809
CKAP4	Cytoskeleton-associated protein 4	+	2.41377	0.0446234	-1.18162
SH3GL1	Endophilin-A2	+	4.12546	0.0238095	-1.15928
CKAP5	Cytoskeleton-associated protein 5	+	3.39884	0.0278974	-1.11194
CANX	Calnexin	+	3.1447	0.0343529	-1.02363
NFKB2	Nuclear factor NF-kappa-B p100 subunit;Nuclear factor NF-ka	+	2.57915	0.0461918	-0.97946
EPS8L2	Epidermal growth factor receptor kinase substrate 8-like pro	+	3.13036	0.0382545	-0.903123
HLA-C		+	2.88459	0.0412	-0.788956
DYNC1H1	Cytoplasmic dynein 1 heavy chain 1	+	2.80611	0.04355	-0.757126
