# Supplementary material for: PDZD8 interacts with Protrudin and Rab7 at ER-late endosome membrane contact sites associated with mitochondria
Source: Nat Commun. 2020 Jul 20;11:3645. doi: 10.1038/s41467-020-17451-7 (PMC7371716; doi:10.1038/s41467-020-17451-7)
Supplement: Supplementary file 3 — Supplementary Dataset 2 [file 41467_2020_17451_MOESM3_ESM.pdf]

Supplementary Data File 2: Potential interactors of Protrudin. Mass-spectrometry based results from an analysis of immunoprecipitates using an anti-Protrudin antibody from crosslinked human HCT116 cell lysates. Significant potential interactors were identified using one-sided Student's t-test (FDR 0.05, S0=0.1)

Gene names	Protein names	Student's T-test Significant Control_ZFYVE27	-Log Student's T-test p-value Control_ZFYVE27	Student's T-test q-value Control_ZFYVE27	Student's T-test Difference Control_ZFYVE27
ZFYVE27	Protrudin	+	5.27168	0	-10.1867
PDZD8	PDZ domain-containing protein 8	+	5.80162	0	-11.5825
VAPB	Vesicle-associated membrane protein-associated protein B/C	+	2.9787	0.00587013	-5.43787
VAPA	Vesicle-associated membrane protein-associated protein A	+	3.21574	0.00627778	-3.05377
MOSPD2	Motile sperm domain-containing protein 2	+	1.97981	0.0160989	-3.37045
RAB7A	Ras-related protein Rab-7a	+	3.14381	0.00607843	-2.23241
KIF5B	Kinesin-1 heavy chain	+	3.05145	0.00647619	-1.79854
OSBP	Oxysterol-binding protein 1	+	3.97549	0	-5.81701
OSBPL3	Oxysterol-binding protein-related protein 3	+	5.95674	0	-6.37125
OSBPL8	Oxysterol-binding protein-related protein 8;Oxysterol-binding protein	+	5.44767	0	-5.38605
RTN4	Reticulon-4;Reticulon	+	1.71603	0.0245375	-3.58816
TRIM21	E3 ubiquitin-protein ligase TRIM21	+	4.33435	0	-9.38908
IGHG1;IGHG3	Ig gamma-1 chain C region;Ig gamma-3 chain C region	+	5.19447	0	-7.78275
COPB1	Coatomer subunit beta	+	2.53186	0.00731507	-5.40086
PRDX3	Thioredoxin-dependent peroxide reductase, mitochondrial	+	2.43729	0.00719512	-5.33494
RAB3GAP1	Rab3 GTPase-activating protein catalytic subunit	+	2.51756	0.00716779	-5.293
DNAJC21	DnaJ homolog subfamily C member 21	+	2.04205	0.012304	-5.2209
GIT1	ARF GTPase-activating protein GIT1	+	2.95008	0.00607059	-5.02645
NBAS	Neuroblastoma-amplified sequence	+	4.134	0	-5.01217
TRIM3	Tripartite motif-containing protein 3	+	3.33784	0.0047619	-4.87881
FNTA	Protein farnesyltransferase/geranylgeranyltransferase type-1 subunit a	+	6.26449	0	-4.80435
ZW10	Centromere/kinetochore protein zw10 homolog	+	1.82743	0.0181141	-4.70503
EIF3F	Eukaryotic translation initiation factor 3 subunit F	+	3.36166	0.005	-4.69428
ACACA	Acetyl-CoA carboxylase 1;Biotin carboxylase	+	2.86209	0.00601942	-4.65085
UBE4A	Ubiquitin conjugation factor E4 A	+	3.72452	0.00176	-4.62364
LONP1	Lon protease homolog, mitochondrial	+	3.6223	0.002875	-4.62246
RAB2A	Ras-related protein Rab-2A	+	3.35766	0.00465116	-4.54571

CCDC186	Coiled-coil domain-containing protein 186	+	2.96556	0.00593103	-4.51385
PLOD1	Procollagen-lysine,2-oxoglutarate 5-dioxygenase 1	+	4.39404	0	-4.39132
STX18	Syntaxin-18	+	2.6622	0.00651515	-4.33164
ESYT1	Extended synaptotagmin-1	+	4.24577	0	-4.25936
FAT1	Protocadherin Fat 1;Protocadherin Fat 1, nuclear form	+	3.67216	0.00317241	-4.2436
ARPC1B	Actin-related protein 2/3 complex subunit 1B	+	2.56308	0.00721622	-4.21334
COPA	Coatomer subunit alpha;Xenin;Proxenin	+	5.66442	0	-4.21082
ESRRA	Steroid hormone receptor ERR1	+	3.14521	0.0061875	-4.20364
ZBTB2	Zinc finger and BTB domain-containing protein 2	+	1.86776	0.0175802	-4.12706
LRCH4	Leucine-rich repeat and calponin homology domain-containing protein	+	2.42564	0.0080226	-4.11944
ACBD5	Acyl-CoA-binding domain-containing protein 5	+	1.92666	0.0161905	-4.07759
REEP6	Receptor expression-enhancing protein 6;Receptor expression-enhanc	+	2.88819	0.00596154	-4.05098
UBXN4	UBX domain-containing protein 4	+	3.47159	0.00365714	-3.99664
NAP1L1	Nucleosome assembly protein 1-like 1	+	2.75488	0.0063	-3.9415
CTPS2	CTP synthase 2	+	2.77136	0.00657391	-3.91849
ATP5I	ATP synthase subunit e, mitochondrial	+	2.52178	0.00698137	-3.9176
STX10	Syntaxin-10	+	3.14926	0.00565714	-3.91311
PROSER2;C10c	Proline and serine-rich protein 2	+	3.16371	0.006	-3.90323
STIM1	Stromal interaction molecule 1	+	3.81644	0.00191304	-3.89908
ITGB4	Integrin beta-4	+	3.42961	0.00512821	-3.88927
EIF3L	Eukaryotic translation initiation factor 3 subunit L	+	2.73662	0.00663415	-3.88127
ANKLE2	Ankyrin repeat and LEM domain-containing protein 2	+	2.99552	0.00579775	-3.86211
SRPR	Signal recognition particle receptor subunit alpha	+	3.35839	0.00392157	-3.83368
VCL	Vinculin	+	3.45238	0.00540541	-3.82928
NFXL1	NF-X1-type zinc finger protein NFXL1	+	3.08739	0.00602667	-3.82695
PLOD3	Procollagen-lysine,2-oxoglutarate 5-dioxygenase 3	+	2.43302	0.00833333	-3.82378
BAG2	BAG family molecular chaperone regulator 2	+	2.21192	0.0115071	-3.82092
ATF6	Cyclic AMP-dependent transcription factor ATF-6 alpha;Processed cycli	+	3.33106	0.00513208	-3.79251
PON1	Serum paraoxonase/arylesterase 1	+	3.02695	0.00614286	-3.79011
ARFGAP2	ADP-ribosylation factor GTPase-activating protein 2	+	2.41307	0.00874317	-3.76255
VPS13C	Vacuolar protein sorting-associated protein 13C	+	2.01818	0.0136952	-3.73673
ATL2	Atlastin-2	+	1.98369	0.0153357	-3.67285
DDX20	Probable ATP-dependent RNA helicase DDX20	+	1.54407	0.0389191	-3.6557

KPNA3	Importin subunit alpha-4	+	2.04793	0.0132424	-3.65437
DHRS7	Dehydrogenase/reductase SDR family member 7	+	3.40301	0.00416667	-3.652
TACC1	Transforming acidic coiled-coil-containing protein 1	+	2.95875	0.00632653	-3.6453
ESYT2	Extended synaptotagmin-2	+	3.38429	0.004	-3.62973
RINT1	RAD50-interacting protein 1	+	2.86459	0.00614545	-3.60313
SCFD1	Sec1 family domain-containing protein 1	+	3.19174	0.00582353	-3.59339
RAB3GAP2	Rab3 GTPase-activating protein non-catalytic subunit	+	2.25452	0.0107767	-3.59242
RBCK1	RanBP-type and C3HC4-type zinc finger-containing protein 1	+	2.72966	0.00661538	-3.57752
MYL3;MYL1	Myosin light chain 3;Myosin light chain 1/3, skeletal muscle isoform	+	2.96086	0.0062	-3.57241
PPFIA1	Liprin-alpha-1	+	3.78088	0.00328571	-3.56671
NUP155	Nuclear pore complex protein Nup155	+	2.09541	0.0123534	-3.55361
MYOF	Myoferlin	+	2.55922	0.00711392	-3.53953
SEPT8	Septin-8	+	3.55697	0.00387879	-3.53182
MIA3	Melanoma inhibitory activity protein 3	+	2.19543	0.011276	-3.51044
CTPS1	CTP synthase 1	+	3.75297	0.00296774	-3.48795
BCAP31	B-cell receptor-associated protein 31	+	2.65237	0.00752113	-3.48737
SDF2L1	Stromal cell-derived factor 2-like protein 1	+	2.9139	0.0064381	-3.48615
KIF21A	Kinesin-like protein KIF21A	+	3.08708	0.00661538	-3.48319
RAB21	Ras-related protein Rab-21	+	1.57524	0.035645	-3.47574
ARPC5	Actin-related protein 2/3 complex subunit 5	+	2.83956	0.00603571	-3.43554
ROCK2	Rho-associated protein kinase 2	+	2.18542	0.0116637	-3.43482
MYH10	Myosin-10	+	2.67695	0.00686131	-3.41997
KTN1	Kinectin	+	4.02159	0.00209524	-3.39667
ITPR3	Inositol 1,4,5-trisphosphate receptor type 3	+	1.80654	0.0208229	-3.37965
CARM1	Histone-arginine methyltransferase CARM1	+	2.59511	0.0072987	-3.36381
DNM1L	Dynamin-1-like protein	+	2.16531	0.0122034	-3.29787
GAPVD1	GTPase-activating protein and VPS9 domain-containing protein 1	+	1.85303	0.0192619	-3.27856
PEX6	Peroxisome assembly factor 2	+	2.3621	0.00879602	-3.26331
HSP90AB2P	Putative heat shock protein HSP 90-beta 2	+	1.9744	0.0164321	-3.24052
ACTR1B	Beta-centractin	+	2.37758	0.00888442	-3.2354
AKAP9	A-kinase anchor protein 9	+	1.94781	0.0164901	-3.2273
CDK5RAP3	CDK5 regulatory subunit-associated protein 3	+	1.7818	0.0224641	-3.21992
SLIRP	SRA stem-loop-interacting RNA-binding protein, mitochondrial	+	2.14327	0.0123636	-3.19683

USE1	Vesicle transport protein USE1	+	1.82717	0.0207188	-3.19607
MYL12B;MYL1	Myosin regulatory light chain 12B;Myosin regulatory light chain 12A	+	1.93979	0.0161688	-3.17174
CEP170	Centrosomal protein of 170 kDa	+	3.29991	0.00453333	-3.17074
MRPL45	39S ribosomal protein L45, mitochondrial	+	1.74101	0.0244444	-3.16703
PRKAR1A	cAMP-dependent protein kinase type I-alpha regulatory subunit;cAMP-	+	3.87364	0.00353846	-3.16131
UFL1	E3 UFM1-protein ligase 1	+	4.3384	0	-3.15291
DNAJB12	DnaJ homolog subfamily B member 12	+	2.1057	0.0122824	-3.15192
OGDH	2-oxoglutarate dehydrogenase, mitochondrial	+	2.79554	0.00658065	-3.14314
HOOK2	Protein Hook homolog 2	+	3.09146	0.006	-3.13275
CYB5R3	NADH-cytochrome b5 reductase 3;NADH-cytochrome b5 reductase 3 n	+	1.68456	0.0282843	-3.12033
BNIP1	Vesicle transport protein SEC20	+	2.75118	0.00656489	-3.11769
STIM2	Stromal interaction molecule 2	+	2.50588	0.00756571	-3.10942
TMOD3	Tropomodulin-3	+	1.6639	0.0297938	-3.10074
PNPLA6	Neuropathy target esterase	+	3.53437	0.00487805	-3.08996
SRP19	Signal recognition particle 19 kDa protein	+	4.2036	0	-3.08827
PSMD1	26S proteasome non-ATPase regulatory subunit 1	+	2.79363	0.0064252	-3.06782
SAR1A;SAR1B	GTP-binding protein SAR1a;GTP-binding protein SAR1b	+	2.15319	0.0124149	-3.06678
STK3	Serine/threonine-protein kinase 3;Serine/threonine-protein kinase 3 3	+	1.80639	0.0215932	-3.06584
HBB	Hemoglobin subunit beta;LVV-hemorphin-7;Spinorphin	+	1.87874	0.0180599	-3.0637
ECM29;KIAA03	Proteasome-associated protein ECM29 homolog	+	1.48178	0.0458856	-3.05656
NDC1	Nucleoporin NDC1	+	1.5137	0.0435051	-2.99867
EDC4	Enhancer of mRNA-decapping protein 4	+	1.79278	0.0225263	-2.9965
SH3GLB2	Endophilin-B2	+	3.90557	0.00340741	-2.98209
OSBPL5	Oxysterol-binding protein-related protein 5;Oxysterol-binding protein	+	2.41038	0.00845128	-2.97838
PSMD7	26S proteasome non-ATPase regulatory subunit 7	+	2.37815	0.00875248	-2.96536
FKBP8	Peptidyl-prolyl cis-trans isomerase FKBP8;Peptidyl-prolyl cis-trans isom	+	1.47852	0.046502	-2.95593
INF2	Inverted formin-2	+	3.34314	0.00468966	-2.94966
PARP4	Poly [ADP-ribose] polymerase 4	+	2.20236	0.0121552	-2.93787
ETF1	Eukaryotic peptide chain release factor subunit 1	+	1.61757	0.03298	-2.93386
SACM1L	Phosphatidylinositide phosphatase SAC1	+	3.28176	0.00609231	-2.93018
AIMP2	Aminoacyl tRNA synthase complex-interacting multifunctional protein	+	2.88612	0.00592982	-2.91924
C1QBP	Complement component 1 Q subcomponent-binding protein, mitocho	+	2.1792	0.0123529	-2.91746
OSBPL9	Oxysterol-binding protein-related protein 9	+	3.54717	0.00454545	-2.91524

VPS35	Vacuolar protein sorting-associated protein 35	+	1.99529	0.0164895	-2.88271
ACTR1A	Alpha-centractin	+	2.54939	0.00740476	-2.86428
MAGT1	Magnesium transporter protein 1	+	2.17226	0.0124667	-2.85183
WDHD1	WD repeat and HMG-box DNA-binding protein 1	+	2.55956	0.00710843	-2.85065
UTP3	Something about silencing protein 10	+	1.5005	0.0449384	-2.83608
SEC24C	Protein transport protein Sec24C	+	1.67725	0.029494	-2.83064
OS9	Protein OS-9	+	1.85648	0.0205782	-2.82414
SPTLC2	Serine palmitoyltransferase 2	+	3.05263	0.00626263	-2.80671
CDKAL1	Threonylcarbamoyladenosine tRNA methylthiotransferase	+	1.89075	0.0185194	-2.76797
ALDH4A1	Delta-1-pyrroline-5-carboxylate dehydrogenase, mitochondrial	+	2.73271	0.00733333	-2.7665
SRP72	Signal recognition particle subunit SRP72	+	1.93688	0.0172956	-2.75615
TOMM70A	Mitochondrial import receptor subunit TOM70	+	1.53791	0.0417988	-2.72163
COPB2	Coatomer subunit beta	+	3.29669	0.00557746	-2.70559
STRAP	Serine-threonine kinase receptor-associated protein	+	1.94717	0.0170978	-2.69601
HOOK1	Protein Hook homolog 1	+	1.81103	0.0224222	-2.68296
SEPT7	Septin-7	+	2.0168	0.0160423	-2.65689
PSMD11	26S proteasome non-ATPase regulatory subunit 11	+	2.88234	0.00635294	-2.654
MRPL11	39S ribosomal protein L11, mitochondrial	+	1.63383	0.0330673	-2.65291
EIF3D	Eukaryotic translation initiation factor 3 subunit D	+	2.58428	0.00715152	-2.64654
PTRF	Polymerase I and transcript release factor	+	2.00715	0.016375	-2.63854
CALU	Calumenin	+	1.7722	0.024384	-2.62469
ACBD3	Golgi resident protein GCP60	+	1.82471	0.021764	-2.62155
NDUFA13	NADH dehydrogenase [ubiquinone] 1 alpha subcomplex subunit 13	+	1.65388	0.0328465	-2.5973
TMEM131	Transmembrane protein 131	+	1.81276	0.0223407	-2.58965
SHMT1	Serine hydroxymethyltransferase, cytosolic	+	3.20212	0.00645	-2.56978
HSD17B4	Peroxisomal multifunctional enzyme type 2;(3R)-hydroxyacyl-CoA dehy	+	2.07092	0.0142125	-2.54888
STRN	Striatin	+	3.14718	0.00608696	-2.52367
ILK	Integrin-linked protein kinase	+	2.11121	0.0134038	-2.52178
SRP54	Signal recognition particle 54 kDa protein	+	3.41153	0.00544262	-2.51485
PSMD13	26S proteasome non-ATPase regulatory subunit 13	+	2.90817	0.00640678	-2.50171
COPG2	Coatomer subunit gamma-2	+	2.04384	0.015516	-2.49766
TNPO3	Transportin-3	+	3.49797	0.00494545	-2.49541
PHRF1	PHD and RING finger domain-containing protein 1	+	2.73452	0.00736552	-2.49333

PPP6R1	Serine/threonine-protein phosphatase 6 regulatory subunit 1	+	2.00226	0.0159732	-2.49331
AKAP11	A-kinase anchor protein 11	+	3.01395	0.00620183	-2.49324
ARHGEF12	Rho guanine nucleotide exchange factor 12	+	2.02938	0.015986	-2.47842
USP9X	Probable ubiquitin carboxyl-terminal hydrolase FAF-X	+	2.59862	0.0074491	-2.47797
DNAJC2	DnaJ homolog subfamily C member 2;DnaJ homolog subfamily C memb	+	1.62512	0.0343668	-2.47787
TRIM4	E3 ubiquitin-protein ligase TRIM4	+	1.64566	0.0328989	-2.47262
CKAP2	Cytoskeleton-associated protein 2	+	1.6571	0.0330278	-2.46726
EIF4E	Eukaryotic translation initiation factor 4E	+	1.76142	0.0249412	-2.46386
ERCC6L	DNA excision repair protein ERCC-6-like	+	1.82663	0.0225475	-2.4627
DDRGK1	DDRGK domain-containing protein 1	+	3.13601	0.00652632	-2.45756
FAM96B	Mitotic spindle-associated MMXD complex subunit MIP18	+	1.87701	0.0205982	-2.45499
SEC22B	Vesicle-trafficking protein SEC22b	+	2.47728	0.00853886	-2.45435
NFAT5	Nuclear factor of activated T-cells 5	+	1.79357	0.0237405	-2.45124
MLLT4	Afadin	+	5.15196	0	-2.43016
SPTLC1	Serine palmitoyltransferase 1	+	3.04295	0.00637736	-2.42133
ERP44	Endoplasmic reticulum resident protein 44	+	3.38462	0.00591045	-2.41427
EEF1B2	Elongation factor 1-beta	+	2.49616	0.00871958	-2.41425
MVP	Major vault protein	+	3.76455	0.00376471	-2.41398
HDAC2	Histone deacetylase 2	+	1.73758	0.02674	-2.40822
SPATA5	Spermatogenesis-associated protein 5	+	3.40898	0.0063871	-2.40468
SLC4A2	Anion exchange protein 2;Anion exchange protein	+	1.76124	0.0252755	-2.4006
PSMD5	26S proteasome non-ATPase regulatory subunit 5	+	1.64808	0.0329933	-2.39989
TMUB1	Transmembrane and ubiquitin-like domain-containing protein 1	+	2.31626	0.0114528	-2.38487
ATXN2	Ataxin-2	+	1.93773	0.0175902	-2.3835
PAWR	PRKC apoptosis WT1 regulator protein	+	1.53371	0.0440162	-2.38022
LMAN1	Protein ERGIC-53	+	3.22711	0.00621687	-2.37968
NOTCH2	Neurogenic locus notch homolog protein 2;Notch 2 extracellular trunca	+	1.62264	0.0352625	-2.37909
MRPS31	28S ribosomal protein S31, mitochondrial	+	1.75549	0.0258734	-2.3732
DNAJB11	DnaJ homolog subfamily B member 11	+	2.80523	0.00691176	-2.37296
ALDH18A1	Delta-1-pyrroline-5-carboxylate synthase;Glutamate 5-kinase;Gamma-	+	2.19704	0.0123592	-2.37044
ARPC2	Actin-related protein 2/3 complex subunit 2	+	1.79807	0.0238167	-2.36987
ASCC2	Activating signal cointegrator 1 complex subunit 2	+	2.60181	0.00765318	-2.34529
LRPPRC	Leucine-rich PPR motif-containing protein, mitochondrial	+	2.30656	0.0111889	-2.34151

DCTN3	Dynactin subunit 3	+	1.54232	0.0435466	-2.33358
NFKB2	Nuclear factor NF-kappa-B p100 subunit;Nuclear factor NF-kappa-B p5[illegible]	+	4.45003	0	-2.31832
CKAP4	Cytoskeleton-associated protein 4	+	2.93822	0.00619672	-2.31277
MT-CO2	Cytochrome c oxidase subunit 2	+	1.98479	0.0165987	-2.30291
CNOT2	CCR4-NOT transcription complex subunit 2	+	3.64756	0.00408163	-2.29873
JPH1	Junctophilin-1	+	2.79618	0.00757447	-2.29837
CSDE1	Cold shock domain-containing protein E1	+	2.72642	0.00744371	-2.2973
COLGALT1	Procollagen galactosyltransferase 1	+	4.37432	0.002	-2.28835
OSBPL10	Oxysterol-binding protein-related protein 10;Oxysterol-binding protein	+	3.07213	0.00631776	-2.28519
EXOC8	Exocyst complex component 8	+	2.587	0.00777273	-2.27594
ACO1	Cytoplasmic aconitate hydratase	+	2.19809	0.0124032	-2.26729
CAPZA2	F-actin-capping protein subunit alpha-2	+	2.14835	0.0131756	-2.26565
MAP1S	Microtubule-associated protein 1S;MAP1S heavy chain;MAP1S light ch	+	1.79086	0.0243799	-2.26346
ATP13A1	Manganese-transporting ATPase 13A1	+	2.2919	0.0118206	-2.25894
P4HA1	Prolyl 4-hydroxylase subunit alpha-1	+	2.19021	0.0122063	-2.25403
TAOK2	Serine/threonine-protein kinase TAO2	+	2.03344	0.0163014	-2.25058
NOP16	Nucleolar protein 16	+	1.81407	0.023458	-2.25032
RELA	Transcription factor p65	+	2.66253	0.00723926	-2.24263
KCMF1	E3 ubiquitin-protein ligase KCMF1	+	2.00542	0.0161165	-2.24186
MSI2	RNA-binding protein Musashi homolog 2	+	2.27308	0.0122078	-2.23962
RAB14	Ras-related protein Rab-14	+	3.79851	0.00355556	-2.23689
TLN1	Talin-1	+	2.49391	0.00869036	-2.23178
PTPN1	Tyrosine-protein phosphatase non-receptor type 1;Tyrosine-protein ph	+	3.04336	0.00609009	-2.22797
EPS8L2	Epidermal growth factor receptor kinase substrate 8-like protein 2	+	1.99739	0.0165385	-2.22768
SCAF1	Splicing factor, arginine/serine-rich 19	+	1.66563	0.0327964	-2.20825
JAKMIP1	Janus kinase and microtubule-interacting protein 1	+	3.74438	0.00444444	-2.20427
ITGB4	Integrin beta-4	+	3.4622	0.00628571	-2.18929
PABPC4	Polyadenylate-binding protein 4;Polyadenylate-binding protein	+	2.82714	0.00768345	-2.16374
UBE2K	Ubiquitin-conjugating enzyme E2 K	+	1.68942	0.0323357	-2.15374
HECTD1	E3 ubiquitin-protein ligase HECTD1	+	1.80449	0.024382	-2.15085
WFS1	Wolframin	+	2.1879	0.0120462	-2.1401
DNAJC1	DnaJ homolog subfamily C member 1	+	2.28312	0.0120513	-2.12196
FASN	Fatty acid synthase;[Acyl-carrier-protein] S-acetyltransferase;[Acyl-carr	+	2.78485	0.00726531	-2.11698

LRRC59	Leucine-rich repeat-containing protein 59	+	1.70196	0.0316432	-2.10998
RAP1B	Ras-related protein Rap-1b;Ras-related protein Rap-1b-like protein	+	1.58729	0.040472	-2.10217
C19orf25	UPF0449 protein C19orf25	+	2.71182	0.00693827	-2.09446
UQCRC1	Cytochrome b-c1 complex subunit 1, mitochondrial	+	1.52401	0.0462441	-2.09421
DCTN1	Dynactin subunit 1	+	3.19234	0.00613861	-2.09313
EIF3K	Eukaryotic translation initiation factor 3 subunit K	+	3.83622	0.00526316	-2.08955
SEPT11	Septin-11	+	2.76781	0.00734641	-2.08865
ATP6V1A	V-type proton ATPase catalytic subunit A	+	1.54032	0.045014	-2.08734
KIF16B	Kinesin-like protein KIF16B	+	1.81612	0.0244492	-2.08438
FAM195A	Protein FAM195A	+	1.67677	0.0329455	-2.08016
MACF1	Microtubule-actin cross-linking factor 1, isoforms 1/2/3/5	+	1.5898	0.04039	-2.0769
SH3GL1	Endophilin-A2	+	4.19169	0.00306667	-2.06948
ATL3	Atlastin-3	+	1.87304	0.0216563	-2.06639
MKL2;MKL1;m	MKL/myocardin-like protein 2;Phosphatase and actin regulator;MKL/m	+	2.6674	0.00783432	-2.05751
VPS4B	Vacuolar protein sorting-associated protein 4B	+	1.80662	0.0244398	-2.05633
CPD	Carboxypeptidase D	+	2.95848	0.006375	-2.05591
RAB6A	Ras-related protein Rab-6A	+	3.29206	0.00586364	-2.04698
ERLIN1	Erlin-1	+	3.23445	0.00645833	-2.04644
RRM1	Ribonucleoside-diphosphate reductase large subunit	+	2.21119	0.0123794	-2.04463
CKAP5	Cytoskeleton-associated protein 5	+	3.63946	0.00485714	-2.04426
FH	Fumarate hydratase, mitochondrial	+	2.09375	0.015461	-2.04217
PDCD2L	Programmed cell death protein 2-like	+	2.04096	0.0164356	-2.04199
SRP68	Signal recognition particle subunit SRP68	+	3.34756	0.00637037	-2.02861
IARS	Isoleucine--tRNA ligase, cytoplasmic	+	3.34368	0.00629268	-2.02613
EEF1E1	Eukaryotic translation elongation factor 1 epsilon-1	+	1.68643	0.0327963	-2.0202
TBK1	Serine/threonine-protein kinase TBK1	+	1.55746	0.0439274	-2.01657
CAPN2	Calpain-2 catalytic subunit	+	1.84195	0.0234239	-2.01629
CD2AP	CD2-associated protein	+	2.30677	0.012103	-2.00518
RPAP3	RNA polymerase II-associated protein 3	+	3.5053	0.00573913	-2.00347
ELP3	Elongator complex protein 3	+	1.90519	0.0209425	-1.99361
CAMSAP1	Calmodulin-regulated spectrin-associated protein 1	+	1.79521	0.0254721	-1.97325
GPD1L	Glycerol-3-phosphate dehydrogenase 1-like protein;Glycerol-3-phosph	+	2.67254	0.0076092	-1.97093
WASF2	Wiskott-Aldrich syndrome protein family member 2	+	2.00393	0.0174161	-1.96508

DDX6	Probable ATP-dependent RNA helicase DDX6	+	1.55957	0.0440562	-1.96183
PRKAA1	5-AMP-activated protein kinase catalytic subunit alpha-1	+	2.39954	0.0109808	-1.9533
LPCAT2	Lysophosphatidylcholine acyltransferase 2	+	2.94969	0.00646617	-1.94958
SEL1L	Protein sel-1 homolog 1	+	2.00767	0.0174704	-1.94943
AP2A1	AP-2 complex subunit alpha-1	+	1.61651	0.0392135	-1.94688
PTRH2	Peptidyl-tRNA hydrolase 2, mitochondrial	+	1.63438	0.0383054	-1.94624
EPS8	Epidermal growth factor receptor kinase substrate 8	+	1.6964	0.033023	-1.94075
RBBP4;RBBP7	Histone-binding protein RBBP4	+	1.85318	0.0234877	-1.94017
DHX29	ATP-dependent RNA helicase DHX29	+	3.76755	0.00523077	-1.93399
RNF214	RING finger protein 214	+	1.97964	0.018006	-1.93142
MYH9	Myosin-9	+	3.09535	0.0059823	-1.92725
GRIPAP1	GRIP1-associated protein 1	+	1.57639	0.0435601	-1.92685
PRDX4	Peroxiredoxin-4	+	2.61745	0.00869565	-1.91824
SARS	Serine--tRNA ligase, cytoplasmic	+	1.78344	0.026807	-1.91541
ATP2C1	Calcium-transporting ATPase type 2C member 1;Calcium-transporting	+	1.90092	0.0211932	-1.91348
SEC24B	Protein transport protein Sec24B	+	1.6647	0.0346957	-1.91096
SEPT2	Septin-2	+	2.99383	0.00632558	-1.9096
COPS5	COP9 signalosome complex subunit 5	+	1.79236	0.0262872	-1.909
EIF3C;EIF3CL	Eukaryotic translation initiation factor 3 subunit C;Eukaryotic translatic	+	3.3459	0.00573333	-1.89825
STX5	Syntaxin-5	+	2.3455	0.0117156	-1.89682
WDR12	Ribosome biogenesis protein WDR12	+	2.00609	0.0174724	-1.89207
REEP4	Receptor expression-enhancing protein 4;Receptor expression-enhanc	+	2.60884	0.00876596	-1.87299
DOCK7	Dedicator of cytokinesis protein 7	+	2.02034	0.017525	-1.87093
NAA15	N-alpha-acetyltransferase 15, NatA auxiliary subunit	+	2.26469	0.0124534	-1.86646
C14orf166	UPF0568 protein C14orf166	+	2.13701	0.0149527	-1.86521
USP19	Ubiquitin carboxyl-terminal hydrolase 19;Ubiquitin carboxyl-terminal h	+	1.73289	0.031799	-1.86133
GRWD1	Glutamate-rich WD repeat-containing protein 1	+	2.5873	0.00858333	-1.85553
ARCN1	Coatomer subunit delta	+	3.8959	0.00425532	-1.85169
SREBF1	Sterol regulatory element-binding protein 1;Processed sterol regulator	+	3.66661	0.00461017	-1.84856
LCLAT1	Lysocardiolipin acyltransferase 1	+	1.53726	0.0464297	-1.84846
DYNC1H1	Cytoplasmic dynein 1 heavy chain 1	+	3.91691	0.00434783	-1.84686
CLPX	ATP-dependent Clp protease ATP-binding subunit clpX-like, mitochond	+	2.0854	0.0162457	-1.84639
RCN1	Reticulocalbin-1	+	2.23413	0.0122759	-1.82601

EIF3G	Eukaryotic translation initiation factor 3 subunit G	+	2.56297	0.00884	-1.81912
ATP5L	ATP synthase subunit g, mitochondrial	+	1.99752	0.0178667	-1.81687
MRPL49	39S ribosomal protein L49, mitochondrial	+	1.9973	0.0179518	-1.79995
ARHGEF10	Rho guanine nucleotide exchange factor 10	+	3.74117	0	-11.3392
COL4A3BP	Collagen type IV alpha-3-binding protein	+	3.95508	0	-6.93357
PITPNM1	Membrane-associated phosphatidylinositol transfer protein 1	+	5.12964	0	-5.74462
BIRC6	Baculoviral IAP repeat-containing protein 6	+	4.00096	0	-5.39135
PPP6R2	Serine/threonine-protein phosphatase 6 regulatory subunit 2	+	1.70022	0.0329196	-1.79796
BRAT1	BRCA1-associated ATM activator 1	+	2.41098	0.0113458	-1.79038
GIT2	ARF GTPase-activating protein GIT2	+	2.16271	0.0146715	-1.78812
GCN1L1	Translational activator GCN1	+	3.00483	0.00641791	-1.77745
MOGS	Mannosyl-oligosaccharide glucosidase	+	2.85686	0.00739474	-1.77176
MEMO1	Protein MEMO1	+	2.86189	0.00749333	-1.76283
TMED9	Transmembrane emp24 domain-containing protein 9	+	1.52249	0.0498301	-1.74258
MTA2	Metastasis-associated protein MTA2	+	1.62407	0.0400832	-1.7414
EIF4G1	Eukaryotic translation initiation factor 4 gamma 1	+	2.60543	0.00873469	-1.73948
CANX	Calnexin	+	3.81529	0.00503704	-1.73887
CAPZA1	F-actin-capping protein subunit alpha-1	+	2.20284	0.013603	-1.73877
TACC2	Transforming acidic coiled-coil-containing protein 2	+	1.93827	0.0208825	-1.73819
RUFY1	RUN and FYVE domain-containing protein 1	+	2.82642	0.00706918	-1.73525
MFN2	Mitofusin-2	+	3.08169	0.006528	-1.72943
CYFIP1;CYFIP2	Cytoplasmic FMR1-interacting protein 1;Cytoplasmic FMR1-interacting	+	3.6007	0.00619178	-1.71812
UBQLN1	Ubiquilin-1	+	2.19385	0.0140221	-1.70714
FNDC3A	Fibronectin type-III domain-containing protein 3A	+	2.3485	0.0124051	-1.70603
VCP	Transitional endoplasmic reticulum ATPase	+	4.62894	0.00183333	-1.70493
KIAA1107	Uncharacterized protein KIAA1107	+	1.84013	0.0250051	-1.70351
MTA1	Metastasis-associated protein MTA1	+	2.04615	0.0173622	-1.69628
ATP5B	ATP synthase subunit beta, mitochondrial;ATP synthase subunit beta	+	1.93869	0.0212536	-1.69184
AGL	Glycogen debranching enzyme;4-alpha-glucanotransferase;Amylo-alph	+	1.64115	0.0394515	-1.68924
MRPL2	39S ribosomal protein L2, mitochondrial	+	2.06901	0.0166603	-1.68333
COPG1	Coatomer subunit gamma-1	+	3.13916	0.00624793	-1.67667
GSPT1	Eukaryotic peptide chain release factor GTP-binding subunit ERF3A	+	2.44038	0.011293	-1.67486
EEF1G	Elongation factor 1-gamma	+	2.94864	0.00746853	-1.67324

ERP29	Endoplasmic reticulum resident protein 29	+	1.91158	0.0224022	-1.67301
PKN2	Serine/threonine-protein kinase N2	+	1.79546	0.0284293	-1.67097
PRRC2A	Protein PRRC2A	+	1.57313	0.0456126	-1.65776
PALLD	Palladin	+	1.68733	0.035568	-1.65565
PPP1CA	Serine/threonine-protein phosphatase PP1-alpha catalytic subunit;Seri	+	1.53641	0.0490213	-1.65195
MMGT1	Membrane magnesium transporter 1	+	1.56181	0.046411	-1.6411
KLC2	Kinesin light chain 2	+	1.73138	0.0327215	-1.6377
TRIOBP	TRIO and F-actin-binding protein	+	2.41093	0.0117679	-1.63452
AGAP3	Arf-GAP with GTPase, ANK repeat and PH domain-containing protein 3	+	1.64273	0.0400502	-1.62796
UBR4	E3 ubiquitin-protein ligase UBR4	+	2.7258	0.00828729	-1.62172
DCTN2	Dynactin subunit 2	+	2.89442	0.00715924	-1.61792
RARS	Arginine--tRNA ligase, cytoplasmic	+	2.86376	0.007025	-1.61579
TNKS1BP1	182 kDa tankyrase-1-binding protein	+	1.61399	0.0434367	-1.60881
SNX1	Sorting nexin-1	+	2.26371	0.0131255	-1.60025
TBC1D15	TBC1 domain family member 15	+	1.90808	0.0232131	-1.60024
ENAH	Protein enabled homolog	+	2.45358	0.0112407	-1.59874
CDH3	Cadherin-3	+	2.70711	0.00860215	-1.59702
KIF3B	Kinesin-like protein KIF3B;Kinesin-like protein KIF3B, N-terminally proc	+	2.31158	0.012255	-1.59684
PXN	Paxillin	+	2.04659	0.0177317	-1.59298
ABI1	Abl interactor 1	+	2.09422	0.0166518	-1.58588
ACTN1	Alpha-actinin-1	+	1.98446	0.020538	-1.5846
RCN2	Reticulocalbin-2	+	1.87044	0.0243523	-1.57215
AHNAK	Neuroblast differentiation-associated protein AHNAK	+	2.22397	0.0139706	-1.56742
HIP1R	Huntingtin-interacting protein 1-related protein	+	1.80601	0.0287767	-1.56674
GEMIN5	Gem-associated protein 5	+	2.42391	0.0117719	-1.56598
SEH1L	Nucleoporin SEH1	+	1.87517	0.0242494	-1.55151
PABPC1;PABP(	Polyadenylate-binding protein 1;Polyadenylate-binding protein;Polyad	+	2.19728	0.0152806	-1.55026
BAG6;BAT3	Large proline-rich protein BAG6	+	3.34188	0.00625926	-1.54311
DNAAF5	Dynein assembly factor 5, axonemal	+	2.40755	0.0122553	-1.54218
TAP2	Antigen peptide transporter 2	+	2.1546	0.0164138	-1.53984
DYNC1LI1	Cytoplasmic dynein 1 light intermediate chain 1	+	2.19302	0.0155714	-1.53429
ANKRD40	Ankyrin repeat domain-containing protein 40	+	1.55819	0.0479845	-1.53424
CALR	Calreticulin	+	2.12141	0.016271	-1.53171

FLNB	Filamin-B	+	2.79038	0.00797753	-1.51755
TRIM47	Tripartite motif-containing protein 47	+	3.23122	0.00646154	-1.51687
VAT1	Synaptic vesicle membrane protein VAT-1 homolog	+	3.7184	0.00610811	-1.51321
PRKCSH	Glucosidase 2 subunit beta	+	2.13757	0.0163816	-1.51294
ABI2	Abl interactor 2	+	1.81137	0.0288696	-1.50696
ARPC4;ARPC4-	Actin-related protein 2/3 complex subunit 4	+	1.8964	0.0244468	-1.50492
UBE2O	E2/E3 hybrid ubiquitin-protein ligase UBE2O	+	1.83609	0.0269181	-1.4954
DYNC1LI2	Cytoplasmic dynein 1 light intermediate chain 2	+	1.73472	0.0332804	-1.49476
IDH3G	Isocitrate dehydrogenase [NAD] subunit gamma, mitochondrial;Isocitra	+	1.8302	0.0277543	-1.49084
KLC1	Kinesin light chain 1	+	2.17494	0.0163183	-1.48359
SP3	Transcription factor Sp3	+	2.16862	0.0163574	-1.48184
HK2	Hexokinase-2;Hexokinase	+	1.72508	0.0345621	-1.48125
AAAS	Aladin	+	2.36323	0.0125041	-1.47871
DARS	Aspartate--tRNA ligase, cytoplasmic	+	3.26118	0.00651724	-1.47313
ZC3H15	Zinc finger CCCH domain-containing protein 15	+	2.38379	0.0124609	-1.46328
PDP1	[Pyruvate dehydrogenase [acetyl-transferring]]-phosphatase 1, mitoch	+	2.16164	0.0163987	-1.45159
IGF2R	Cation-independent mannose-6-phosphate receptor	+	1.76663	0.0330993	-1.44952
CCDC47	Coiled-coil domain-containing protein 47	+	2.45325	0.0116696	-1.44491
ASPH	Aspartyl/asparaginyl beta-hydroxylase	+	2.54284	0.0109282	-1.44395
USP10	Ubiquitin carboxyl-terminal hydrolase 10	+	2.03713	0.020213	-1.44237
MAPRE1	Microtubule-associated protein RP/EB family member 1	+	2.50553	0.0111376	-1.44181
HSP90B1	Endoplasmin	+	2.98454	0.00720513	-1.43724
EIF3I	Eukaryotic translation initiation factor 3 subunit I	+	2.38815	0.0124098	-1.4358
PRKAR2A	cAMP-dependent protein kinase type II-alpha regulatory subunit	+	3.54853	0.00639175	-1.43411
ACTR3	Actin-related protein 3	+	2.34588	0.0121395	-1.42976
UNC45A	Protein unc-45 homolog A	+	1.79503	0.0315671	-1.4278
ANKHD1	Ankyrin repeat and KH domain-containing protein 1	+	1.66666	0.0406021	-1.41253
PKMYT1	Membrane-associated tyrosine- and threonine-specific cdc2-inhibitory	+	1.927	0.0242151	-1.4125
NARS	Asparagine--tRNA ligase, cytoplasmic	+	1.74607	0.0339207	-1.40771
CLIP2	CAP-Gly domain-containing linker protein 2	+	3.60175	0.00659574	-1.40392
ATP6V1B2	V-type proton ATPase subunit B, brain isoform	+	1.76827	0.0328707	-1.40232
PSMB2	Proteasome subunit beta type-2	+	2.08995	0.0176778	-1.40169
PSMC4	26S protease regulatory subunit 6B	+	2.74674	0.00849485	-1.39784

IKBKG	NF-kappa-B essential modulator	+	2.89497	0.00769767	-1.39029
PIGK	GPI-anchor transamidase	+	1.90702	0.0248351	-1.38948
SPATA5L1	Spermatogenesis-associated protein 5-like protein 1	+	1.6755	0.0403884	-1.38057
RANBP1	Ran-specific GTPase-activating protein	+	2.80055	0.00855615	-1.37636
NCKAP1	Nck-associated protein 1	+	2.11998	0.0175262	-1.36804
RUVBL1	RuvB-like 1	+	4.12588	0.00477193	-1.36656
COG1	Conserved oligomeric Golgi complex subunit 1	+	1.80359	0.0322342	-1.36465
ITGB1	Integrin beta-1	+	1.68652	0.0401002	-1.3553
PSMG1	Proteasome assembly chaperone 1	+	1.87063	0.0269851	-1.34572
TXLNA	Alpha-taxilin	+	1.61125	0.0461532	-1.34213
SNX2	Sorting nexin-2	+	3.21089	0.00696296	-1.34155
ACAA1	3-ketoacyl-CoA thiolase, peroxisomal	+	2.05077	0.0206588	-1.33866
ARHGAP4	Rho GTPase-activating protein 4	+	1.89052	0.0266231	-1.32906
NUFIP2	Nuclear fragile X mental retardation-interacting protein 2	+	1.76167	0.0343947	-1.32318
GRSF1	G-rich sequence factor 1	+	1.68253	0.0408889	-1.31439
EEF1D	Elongation factor 1-delta	+	2.32669	0.0137463	-1.30776
ZMPSTE24	CAAX prenyl protease 1 homolog	+	2.72348	0.00898522	-1.30513
TBL2	Transducin beta-like protein 2	+	2.20262	0.0162745	-1.28975
SUN2	SUN domain-containing protein 2	+	3.19742	0.00762857	-1.28712
RRBP1	Ribosome-binding protein 1	+	1.94146	0.024376	-1.28466
PDLIM5	PDZ and LIM domain protein 5	+	1.70521	0.0400335	-1.2815
PDCD5	Programmed cell death protein 5	+	2.18065	0.016962	-1.27096
TRAP1	Heat shock protein 75 kDa, mitochondrial	+	2.79104	0.00892929	-1.27043
ACSL3	Long-chain-fatty-acid--CoA ligase 3	+	1.93491	0.0249152	-1.27042
SNX5	Sorting nexin-5	+	2.83913	0.00867368	-1.26844
ACSL5	Long-chain-fatty-acid--CoA ligase 5	+	2.57038	0.0110364	-1.2668
CLNS1A	Methylosome subunit pICln	+	2.01324	0.0229589	-1.24137
TRIM25	E3 ubiquitin/ISG15 ligase TRIM25	+	2.3692	0.0136541	-1.23552
INPPL1	Phosphatidylinositol 3,4,5-trisphosphate 5-phosphatase 2	+	2.11169	0.0193947	-1.23517
PDHA1	Pyruvate dehydrogenase E1 component subunit alpha, somatic form, r	+	2.66165	0.0107246	-1.23303
IMPDH2	Inosine-5-monophosphate dehydrogenase 2	+	1.75485	0.0371121	-1.23053
MYH14	Myosin-14	+	3.79781	0.00666667	-1.2225
RAB11FIP1	Rab11 family-interacting protein 1	+	2.31968	0.0148986	-1.22146

TMCO1	Transmembrane and coiled-coil domain-containing protein 1	+	1.7884	0.034442	-1.21978
FAM98A	Protein FAM98A	+	1.75147	0.0382232	-1.21823
CAD	CAD protein;Glutamine-dependent carbamoyl-phosphate synthase;Asp	+	2.74466	0.00986275	-1.20871
FLNC	Filamin-C	+	3.02369	0.00774269	-1.20754
LARS	Leucine--tRNA ligase, cytoplasmic	+	2.937	0.00879121	-1.20445
PSMD2	26S proteasome non-ATPase regulatory subunit 2	+	2.09218	0.0206512	-1.20388
VARS	Valine--tRNA ligase	+	2.73927	0.0101268	-1.20332
WDR55	WD repeat-containing protein 55	+	1.65495	0.0452698	-1.20279
SORD	Sorbitol dehydrogenase	+	4.02391	0.00594737	-1.19861
EIF3E	Eukaryotic translation initiation factor 3 subunit E	+	1.79745	0.0338462	-1.19808
MRPS23	28S ribosomal protein S23, mitochondrial	+	2.25602	0.01628	-1.19759
AARS	Alanine--tRNA ligase, cytoplasmic	+	2.26271	0.0160269	-1.19597
SOAT1	Sterol O-acyltransferase 1	+	2.67901	0.0108762	-1.18116
UQCRH	Cytochrome b-c1 complex subunit 6, mitochondrial	+	1.81893	0.0329067	-1.1695
PSMD3	26S proteasome non-ATPase regulatory subunit 3	+	1.87218	0.031715	-1.15855
PSMC1	26S protease regulatory subunit 4	+	2.07683	0.0216544	-1.15543
FLII	Protein flightless-1 homolog	+	1.93463	0.0270524	-1.15461
TPD52L2	Tumor protein D54	+	2.67076	0.0113991	-1.15399
PSME1	Proteasome activator complex subunit 1	+	1.99265	0.0245039	-1.14519
CCT8	T-complex protein 1 subunit theta	+	4.04882	0.00653165	-1.14412
WDR1	WD repeat-containing protein 1	+	1.92607	0.0278227	-1.14253
MYL6	Myosin light polypeptide 6	+	1.91249	0.0289395	-1.13406
SMN1;SMN2	Survival motor neuron protein	+	1.75702	0.0392966	-1.13215
IPO5	Importin-5	+	2.281	0.0163279	-1.12858
EIF3H	Eukaryotic translation initiation factor 3 subunit H	+	2.1263	0.0207114	-1.12841
ARHGEF1	Rho guanine nucleotide exchange factor 1	+	2.01554	0.0245147	-1.12725
ATXN2L	Ataxin-2-like protein	+	1.71296	0.0434029	-1.12534
HLA-C		+	3.09914	0.00778824	-1.12505
SPAG9	C-Jun-amino-terminal kinase-interacting protein 4	+	2.29706	0.0159197	-1.1244
RLTPR	Leucine-rich repeat-containing protein 16C	+	1.93923	0.0272277	-1.12188
EMD	Emerin	+	2.49534	0.0121868	-1.1198
RAB8A	Ras-related protein Rab-8A	+	1.88676	0.0317905	-1.11936
ANXA2;ANXA2	Annexin A2;Annexin;Putative annexin A2-like protein	+	3.32443	0.00741667	-1.11346

IRS1	Insulin receptor substrate 1	+	1.92719	0.0288467	-1.11252
CLTA	Clathrin light chain A	+	1.68656	0.0449243	-1.108
MRPL15	39S ribosomal protein L15, mitochondrial	+	3.05796	0.00793296	-1.10781
EPRS	Bifunctional glutamate/proline--tRNA ligase;Glutamate--tRNA ligase;Pr	+	2.12287	0.0209364	-1.10501
PSME2	Proteasome activator complex subunit 2	+	2.42341	0.0140741	-1.09614
ASNS	Asparagine synthetase [glutamine-hydrolyzing]	+	2.64264	0.0117205	-1.09338
CCT6A	T-complex protein 1 subunit zeta	+	1.75534	0.0401667	-1.09096
MPP7	MAGUK p55 subfamily member 7	+	1.69405	0.045104	-1.08806
HDLBP	Vigilin	+	2.67116	0.0116123	-1.07583
NSF	Vesicle-fusing ATPase	+	2.7086	0.0110868	-1.06745
AKAP12	A-kinase anchor protein 12	+	2.70003	0.0115135	-1.06176
DNAJA2	DnaJ homolog subfamily A member 2	+	1.86096	0.0330354	-1.04921
HSP90AB1	Heat shock protein HSP 90-beta	+	4.13742	0.00615385	-1.04669
PSMB5	Proteasome subunit beta type-5	+	2.35072	0.0160811	-1.04211
ATP5H	ATP synthase subunit d, mitochondrial	+	1.7434	0.0434918	-1.03928
AMPD2	AMP deaminase 2	+	1.67241	0.0478677	-1.0289
PSME3	Proteasome activator complex subunit 3	+	2.17018	0.0210029	-1.01425
CRLF3	Cytokine receptor-like factor 3	+	1.81055	0.0388365	-1.01088
RAP1GDS1	Rap1 GTPase-GDP dissociation stimulator 1	+	1.93556	0.0318663	-1.00615
MTHFD1L	Monofunctional C1-tetrahydrofolate synthase, mitochondrial	+	2.44222	0.0156272	-1.00002
LIMD1	LIM domain-containing protein 1	+	1.79591	0.0401176	-0.998254
DNAJA1	DnaJ homolog subfamily A member 1	+	1.96719	0.0294231	-0.986506
EHBP1L1	EH domain-binding protein 1-like protein 1	+	1.83076	0.0385011	-0.981099
UBE4B	Ubiquitin conjugation factor E4 B	+	1.76559	0.0435935	-0.971836
CCT5	T-complex protein 1 subunit epsilon	+	2.62391	0.0122344	-0.959883
PPP2R1A	Serine/threonine-protein phosphatase 2A 65 kDa regulatory subunit A	+	3.19401	0.00864865	-0.946864
MRPL18	39S ribosomal protein L18, mitochondrial	+	2.18294	0.0217031	-0.938083
MARS	Methionine--tRNA ligase, cytoplasmic	+	2.17323	0.0224847	-0.937227
PSMC5	26S protease regulatory subunit 8	+	2.40675	0.0162215	-0.934537
IQGAP1	Ras GTPase-activating-like protein IQGAP1	+	2.74422	0.0125188	-0.93216
ZC3HAV1	Zinc finger CCCH-type antiviral protein 1	+	2.03386	0.0284988	-0.917403
CTTN	Src substrate cortactin	+	1.95137	0.0329471	-0.911373
SND1	Staphylococcal nuclease domain-containing protein 1	+	2.12511	0.0243158	-0.903797

ADAM15	Disintegrin and metalloproteinase domain-containing protein 15	+	2.09809	0.025374	-0.90143
KARS	Lysine--tRNA ligase	+	2.39364	0.0172414	-0.895737
FLNA		+	2.48306	0.0161356	-0.882753
DHX30	Putative ATP-dependent RNA helicase DHX30	+	2.01178	0.0316398	-0.874439
P4HB	Protein disulfide-isomerase	+	2.11115	0.0259596	-0.86462
CTNNA1	Catenin alpha-1	+	1.97135	0.0328288	-0.86384
ARHGEF2	Rho guanine nucleotide exchange factor 2	+	2.749	0.0120927	-0.846774
UPF1	Regulator of nonsense transcripts 1	+	1.81078	0.0444008	-0.84189
TRIP10	Cdc42-interacting protein 4	+	2.4995	0.0162186	-0.834333
HSP90AA1	Heat shock protein HSP 90-alpha	+	2.78856	0.0123307	-0.828227
STT3B	Dolichyl-diphosphooligosaccharide--protein glycosyltransferase subuni	+	2.05717	0.0314906	-0.813467
NEMF	Nuclear export mediator factor NEMF	+	1.81927	0.0456079	-0.803697
REEP3	Receptor expression-enhancing protein 3	+	2.0303	0.0328716	-0.800393
TCP1	T-complex protein 1 subunit alpha	+	3.47122	0.00862827	-0.778273
PSMC3	26S protease regulatory subunit 6A	+	1.96506	0.0390021	-0.76195
SH3KBP1	SH3 domain-containing kinase-binding protein 1	+	1.81994	0.0478519	-0.749034
PDLIM1	PDZ and LIM domain protein 1	+	2.07832	0.0329112	-0.743907
CCT3	T-complex protein 1 subunit gamma	+	2.13233	0.031565	-0.735797
PSMA5	Proteasome subunit alpha type-5	+	2.0719	0.0329667	-0.734027
POLR2B	DNA-directed RNA polymerase II subunit RPB2;DNA-directed RNA poly	+	4.13905	0.00725161	-0.717764
RUVBL2	RuvB-like 2	+	1.93976	0.043839	-0.690264
PDIA6	Protein disulfide-isomerase A6	+	2.04256	0.038641	-0.690016
NMT1	Glycylpeptide N-tetradecanoyltransferase 1	+	2.17904	0.0329374	-0.67009
ATP5O	ATP synthase subunit O, mitochondrial	+	2.44563	0.0243125	-0.629454
TXNDC12	Thioredoxin domain-containing protein 12	+	2.09639	0.0402021	-0.624037
HSPA5	78 kDa glucose-regulated protein	+	2.50826	0.0278914	-0.558083
PSMC6	26S protease regulatory subunit 10B	+	2.58952	0.0328036	-0.480863
DPYSL2	Dihydropyrimidinase-related protein 2	+	2.68274	0.0321589	-0.472036
PRDX1	Peroxiredoxin-1	+	2.29788	0.0491163	-0.44853
