# Supplementary material for: PDZD8 interacts with Protrudin and Rab7 at ER-late endosome membrane contact sites associated with mitochondria
Source: Nat Commun. 2020 Jul 20;11:3645. doi: 10.1038/s41467-020-17451-7 (PMC7371716; doi:10.1038/s41467-020-17451-7)
Supplement: Supplementary file 8 — Reporting Summary [file 41467_2020_17451_MOESM8_ESM.pdf]

nature research

Corresponding author(s): Prof. Jodi Nunnari
Dr. Yael Elbaz-Alon

Last updated by author(s): Jun 3, 2020

Reporting Summary

Nature Research wishes to improve the reproducibility of the work that we publish. This form provides structure for consistency and transparency in reporting. For further information on Nature Research policies, see Authors & Referees and the Editorial Policy Checklist.

Statistics

For all statistical analyses, confirm that the following items are present in the figure legend, table legend, main text, or Methods section.

n/a	Confirmed	
☐	☒	The exact sample size (*n*) for each experimental group/condition, given as a discrete number and unit of measurement
☐	☒	A statement on whether measurements were taken from distinct samples or whether the same sample was measured repeatedly
☐	☒	The statistical test(s) used AND whether they are one- or two-sided<br>*Only common tests should be described solely by name; describe more complex techniques in the Methods section.*
☒	☐	A description of all covariates tested
☐	☒	A description of any assumptions or corrections, such as tests of normality and adjustment for multiple comparisons
☐	☒	A full description of the statistical parameters including central tendency (e.g. means) or other basic estimates (e.g. regression coefficient) AND variation (e.g. standard deviation) or associated estimates of uncertainty (e.g. confidence intervals)
☐	☒	For null hypothesis testing, the test statistic (e.g. *F*, *t*, *r*) with confidence intervals, effect sizes, degrees of freedom and *P* value noted<br>*Give P values as exact values whenever suitable.*
☒	☐	For Bayesian analysis, information on the choice of priors and Markov chain Monte Carlo settings
☒	☐	For hierarchical and complex designs, identification of the appropriate level for tests and full reporting of outcomes
☒	☐	Estimates of effect sizes (e.g. Cohen's *d*, Pearson's *r*), indicating how they were calculated

Our web collection on statistics for biologists contains articles on many of the points above.

Software and code

Policy information about availability of computer code

Data collection	Mass spectrometry data were acquired using the Xcalibur software (Thermo Fisher Scientific).
Data analysis	As stated in the methods section: Raw mass spectrometry files were analyzed with MaxQuant version 1.5.6.9 and the Andromeda search engine. All statistical tests and calculations were done using the Perseus software version 1.5.6.0.<br>Both are publicly available packages: MaxQuant is freely available at https://maxquant.net/maxquant/.<br>Andromeda search engine is implemented in MaxQuant package.<br>Perseus is freely available at https://maxquant.net/perseus/<br>Live cell imaging was performed using VisiScope Confocal Cell Explorer system controlled by VisView software version 3.2.0.0 (Visitron Systems GmbH). If necessary, slight linear adjustments to contrast and brightness were made using ImageJ 1.52v (NIH).<br>For CLEM experiments tomograms were reconstructed using IMOD software package (versions 4.9.4)<br>Analysis in supplementary figure 6 was performed using Imaris Cell Imaging software (OXFORD Instruments) v9.2.1

For manuscripts utilizing custom algorithms or software that are central to the research but not yet described in published literature, software must be made available to editors/reviewers. We strongly encourage code deposition in a community repository (e.g. GitHub). See the Nature Research guidelines for submitting code & software for further information.

Data

Policy information about availability of data

All manuscripts must include a data availability statement. This statement should provide the following information, where applicable:

- Accession codes, unique identifiers, or web links for publicly available datasets
- A list of figures that have associated raw data
- A description of any restrictions on data availability

Mass spectrometry analysis results for the interactomes of PDZD8 and Protrudin are provided with the textmanuscript as supplementary Data file 1 and 2. The

proteomic datasets that support these analyses are available in the PRoteomics IDEntifications (PRIDE) database .Data are available via ProteomeXchange with identifier PXD015523. https://www.ebi.ac.uk/pride/archive/projects/PXD015523. The source data underlying Figures 1A, 1B, 2D, 2F, 4B and Supplementary Figures 1,2,4 and 7 are provided as a Source Data file. Other data are available from the corresponding author upon reasonable request.

Field-specific reporting

Please select the one below that is the best fit for your research. If you are not sure, read the appropriate sections before making your selection.

- [x] Life sciences
- [ ] Behavioural & social sciences
- [ ] Ecological, evolutionary & environmental sciences

For a reference copy of the document with all sections, see nature.com/documents/nr-reporting-summary-flat.pdf

Life sciences study design

All studies must disclose on these points even when the disclosure is negative.

Sample size	For proteomic analyses we routinely use three biological replicates which enables to run solid statistical tests. Results in supplementary data files 1 and 2 reflect interactions that are reproducible in all three immunoprecipitation experiments.<br>PDZD8-GFP/mCherry was imaged in over 70 independent live-cell imaging sessions, Protrudin-GFP/mCherry was imaged in 55 different sessions and the different PDZD8 truncated constructs were each imaged between 4 to 25 different experiments. All images present cells that are loyal representative of the relevant population.
Data exclusions	No data was excluded.
Replication	For mass spectrometry analyses: Three biological replicates were prepared for each sample group. Results provided in supplementary data files 1 and 2 reflect interactions that are reproducible in all three immunoprecipitation experiments. Mass Spectrometry analysis results were also recapitulated through coIP experiments.<br>All imaging experiments were replicated successfully.
Randomization	Not relevant to this study because cells/samples were analyzed in the same way.
Blinding	Blinding was not relevant to this study because cells/samples were analyzed in the same way.

Reporting for specific materials, systems and methods

We require information from authors about some types of materials, experimental systems and methods used in many studies. Here, indicate whether each material, system or method listed is relevant to your study. If you are not sure if a list item applies to your research, read the appropriate section before selecting a response.

Materials & experimental systems

n/a	Involved in the study	
[ ]	[x]	Antibodies
[ ]	[x]	Eukaryotic cell lines
[x]	[ ]	Palaeontology
[x]	[ ]	Animals and other organisms
[x]	[ ]	Human research participants
[x]	[ ]	Clinical data

Methods

n/a	Involved in the study	
[x]	[ ]	ChIP-seq
[x]	[ ]	Flow cytometry
[x]	[ ]	MRI-based neuroimaging

Antibodies

Antibodies used	Primary antibodies used:<br>anti-Actin from Abcam (Cat. No. Ab8224)<br>anti-PDZD8 was a generous gift from Prof. Joseph Sodroski (reference: Zhang et al. 2015. PMID:25771112)<br>anti-Protrudin from ProteinTech (Cat. No. 12680-1-AP)<br>anti-GFP from Abcam (Cat. No. Ab290)<br>anti-Rab7 from Cell Signaling Technology (Cat no. D95F2)<br>Secondary antibodies used:<br>Goat anti-Rabbit IgG H&L 680 from Abcam (Cat. no. Ab216777)<br>Goat anti-Mouse IgG H&L 680 from Abcam (Cat. no. Ab216776)<br>IRDye 800CW Goat anti-Rabbit IgG from LI-COR Biosciences (Cat. no. 926-3211)<br>IRDye 680RD Goat anti-mouse IgG from LI-COR Biosciences (Cat. No. 926-68070)<br>Alexa Fluor 488 goat anti-Rabbit from ThermoFisher Scientific (Ca. no. A-11034)<br>Alexa Fluor 568 goat anti-Mouse from ThermoFisher Scientific (Ca. no. A-11031)

Validation

Anti-PDZD8: Zhang et al. 2015. PMID:25771112
For both anti-PDZD8 and anti-Protrudin, mass -spectrometry results validate the antibodies target.
All commercial antibodies are widely used common antibodies. Anti-GFP (Abcam Ab290), Anti-Rab7 (CST D95F2) and anti-Actin (Abcam Ab8224) were validated by manufacturer and supported by multiple publications.

Eukaryotic cell lines

Policy information about cell lines

Cell line source(s)

Cell lines used and obtained from ATCC:
U2OS (U-2 OS; ATCC® HTB-96)
COS7 (COS-7; ATCC® CRL-1651)
HCT116 (HCT 116; ATCC® CCL-247™)
HEK293T (HEK 293T/17; ATCC® CRL-11268™)
The human fibroblasts are derived from human skin biopsy of a healthy subject. The cell line was received from Prof. Orly Elpeleg (Department of Genetic Research, Hadassah-Hebrew University Medical Center, Jerusalem, Israel) and appear as the control sample in: Edvardson, S., Elbaz-Alon, Y., Jalas, C. et al. Neurogenetics (2016) 17: 219. https://doi.org/10.1007/s10048-016-0487-z.

Authentication

All cell lines present the characteristic morphology. No further authentication procedure was performed.

Mycoplasma contamination

All cell lines were tested negative for Mycoplasma contamination.

Commonly misidentified lines
(See ICLAC register)

None.
